# Blockage of bacterial FimH prevents mucosal inflammation associated with Crohn’s disease

**DOI:** 10.1186/s40168-021-01135-5

**Published:** 2021-08-23

**Authors:** Grégoire Chevalier, Arnaud Laveissière, Guillaume Desachy, Nicolas Barnich, Adeline Sivignon, Marc Maresca, Cendrine Nicoletti, Eric Di Pasquale, Margarita Martinez-Medina, Kenneth William Simpson, Vijay Yajnik, Harry Sokol, MOBIDIC Study Investigators, Temitayo Adegbamigbe, Tariq Ahmad, Ian Arnott, Yoram Bouhnik, Franck Carbonnel, Jean-Frédéric Colombel, Glen Doherty, J. R. Fraser Cummings, Xavier Hébuterne, Hans Herfarth, David Kevans, Guillaume Pineton de Chambrun, Maria Nachury, Stéphane Nancey, Xavier Roblin, Mark A. W. Tremelling, Jonathan Plassais, Francesco Strozzi, Alessandra Cervino, Rachel Morra, Christophe Bonny

**Affiliations:** 1 Enterome, 94-96 Avenue Ledru-Rollin, 75011 Paris, France; 2 Université Clermont Auvergne, Inserm U1071, M2iSH, USC-INRA 2018, F-63000 Clermont-Ferrand, France; 3 Aix Marseille Université, CNRS, Centrale Marseille, iSm2, Marseille, France; 4 Aix-Marseille Université, CNRS, INP, Institut de Neurophysiopathologie, Marseille, France; 5 Microbiology of Intestinal Diseases, Biology Department, Universitat de Girona, Girona, Spain; 6 College of Veterinary Medicine, Cornell University, Ithaca, NY 14853 USA; 7 GI Therapeutic Area Unit, Takeda Pharmaceuticals, Cambridge, MA 02139 USA; 8 Gastroenterology Department, Sorbonne Université, INSERM, Centre de Recherche Saint-Antoine, CRSA, AP-HP, Saint Antoine Hospital, 75012 Paris, France; 9 INRA, UMR1319 Micalis & AgroParisTech, Jouy en Josas, France; 10 Paris Center for Microbiome Medicine (PaCeMM) FHU, AP-HP, Paris, France

**Keywords:** Crohn’s disease, Inflammation, FimH, *Enterobacteriaceae*

## Abstract

**Background:**

An *Escherichia coli* (*E. coli*) pathotype with invasive properties, first reported by Darfeuille-Michaud and termed adherent-invasive *E. coli* (AIEC), was shown to be prevalent in up to half the individuals with Crohn’s Disease (CD), suggesting that these bacteria could be involved in the pathophysiology of CD. Among the genes related to AIEC pathogenicity, *fim* has the potential to generate an inflammatory reaction from the intestinal epithelial cells and macrophages, as it interacts with TLR4, inducing the production of inflammatory cytokines independently of LPS. Therefore, targeting the bacterial adhesion of FimH-expressing bacteria seems a promising therapeutic approach, consisting of disarming bacteria without killing them, representing a selective strategy to suppress a potentially critical trigger of intestinal inflammation, without disturbing the intestinal microbiota.

**Results:**

We analyzed the metagenomic composition of the gut microbiome of 358 patients with CD from two different cohorts and characterized the presence of FimH-expressing bacteria. To assess the pathogenic role of FimH, we used human intestinal explants and tested a specific FimH blocker to prevent bacterial adhesion and associated inflammation. We observed a significant and disease activity-dependent enrichment of *Enterobacteriaceae* in the gut microbiome of patients with CD. Bacterial FimH expression was functionally confirmed in ileal biopsies from 65% of the patients with CD. Using human intestinal explants, we further show that FimH is essential for adhesion and to trigger inflammation. Finally, a specific FimH-blocker, TAK-018, inhibits bacterial adhesion to the intestinal epithelium and prevents inflammation, thus preserving mucosal integrity.

**Conclusions:**

We propose that TAK-018, which is safe and well tolerated in humans, is a promising candidate for the treatment of CD and in particular in preventing its recurrence.

Video abstract

**Supplementary Information:**

The online version contains supplementary material available at 10.1186/s40168-021-01135-5.

## Background

Crohn’s disease (CD) is a chronic inflammatory disease of the gastrointestinal (GI) tract involving the dynamic interaction of host’s genetics, microbiome, and inflammatory responses [1]. Compelling evidence suggests that the microbiome plays a significant role in triggering an abnormal mucosal immune response in patients with CD [2]. An *Escherichia coli* (*E. coli*) pathotype with invasive properties, first reported by Darfeuille-Michaud [3] and termed adherent-invasive *E. coli* (AIEC) [4], was shown to be prevalent in up to half the individuals with CD [5–9], suggesting that these bacteria could be involved in the pathophysiology of CD.

Besides their ability to adhere and invade, AIECs can also survive and replicate in intestinal epithelial cells and macrophages, stimulating the production of inflammatory cytokines [2, 10, 11]. The presence of non-classic virulence factors of adherence and invasion distinguishes AIECs from other *E. coli* strains [12–14]. Interestingly, the genetic factors that are characteristic of the AIEC pathotype are still unknown, as the majority of genes related to their pathogenicity are present commonly in both commensal and pathogenic *E. coli* strains, suggesting that pathoadaptive evolution is an important determinant of *E. coli* pathogenicity. Among these genes, *FimH* codes for a mannose-binding adhesin presented at the tip of type 1 pili, expressed by pathogenic *E.coli* and members of the *Enterobacteriaceae* family [15–18], that allows them to recognize and binds terminal mannoses on epithelial glycoproteins [19–21], including carcinoembryonic antigen-related cell adhesion molecule 6 (CEACAM6) [19] and Toll-like receptor 4 (TLR4) [16, 17]. Even though not reported specifically, other mannosylated abundant components like mucus might also provide high-capacity substrates for FimH attachment [22], allowing for biofilm formation and bacteria-specific mucosal immune responses [12, 15, 19, 21, 23]. Despite their role as sources of pro-inflammatory cytokines [24], normal healthy intestinal epithelial cells (IECs), as well as intestinal macrophages lack the TLR4-accessory proteins CD14 or MD2, rendering these cell types resistant to lipopolysaccharides (LPS)-induced inflammation [25–32]. In particular, resident intestinal macrophages in non-inflamed mucosa have been described as being inflammation “adverse” or “anergic” and will receive the assistance of newly recruited circulating monocytes when needs arise [27]. In sharp contrast, the CD14/MD2-lacking TLR4 in IECs and intestinal macrophages remain sensitive to the action of FimH. In turn, FimH, which is expressed by the type of pathogenic *E. coli* reported to be found in the GI track of patients with CD, has the potential to generate an inflammatory reaction from the intestinal epithelial cells and macrophages residing in (so far) non-inflamed mucosa. Type 1 pili-mediated adhesion to host cells is a crucial step in the establishment of *E. coli* adherence and subsequent invasive process [33, 34]. These mechanisms set then the stage for a selective over colonization of the epithelium by AIECs, with subsequent biofilm formation and bacteria-specific mucosal immune responses [12, 15, 19, 21, 23]. FimH serves as a trigger of inflammation via its interaction with TLR4, inducing the production of tumor necrosis factor alpha (TNFα), interleukin 6 (IL-6) and IL-8 in the gut, independently of LPS [16]. The critical role of FimH as a pro-inflammatory mediator in CD stresses the importance of designing therapeutic strategies that can disrupt this pathogenic pathway [35].

Here, we report the metagenomic analyses of fecal samples from two different cohorts of patients with CD, representing 358 patients, compared with 43 healthy volunteers. Using unbiased shotgun sequencing, we show that *E. coli*, which represents a moderately abundant species in healthy individuals, becomes a quantitatively important species in CD fecal samples. Our data indicate that FimH-expressing bacteria might significantly contribute to the inflammatory process in CD and confer strong support for pharmacological inhibition of FimH in patients with CD. Accordingly, the FimH-blocker TAK-018, a safe and tolerated clinical candidate, might selectively help disarm and clear the harmful FimH-expressing bacteria without directly interfering with the other members of the microbial community. Its mode of action and its expected low impact on the microbiome will clearly avoid many of the generally unacceptable complications and limitations that chronic antibiotic treatments display.

## Methods

### Population for metagenomic study

The first cohort of patients with CD comes from the CrohnOmeter study (Enterome-sponsored) which was an exploratory longitudinal study, conducted at the Saint-Antoine and Saint-Louis Hospitals (Paris, France) and at the French patient association l’Association François Aupetit (AFA, Paris, France). Patients were followed longitudinally for about 9 months, providing monthly stool samples collected at home. For each sample, patients filled a questionnaire, and the disease activity was assessed by the Harvey-Bradshaw index (HBI) [36] and fecal calprotectin [37]. As the *E. coli* abundance and HBI remain stable throughout time (Supplementary Fig1a, b), for the sake of clarity, only the first time point available for each patient is depicted in figures.

The second cohort of patients with CD comes from the PREDICT study (AbbVie sponsored) which was a multicenter, global, cross-sectional, non-interventional study. A total of 305 patients with CD were enrolled into the study, of which 284 patients provided a stool sample before an ileocolonoscopy. Relevant clinical information such as HBI, calprotectin, C-reactive protein (CRP), and endoscopic scores were monitored.

The third cohort of patients with CD comes from the multicenter, international MOBIDIC descriptive study (Enterome-sponsored) which was a longitudinal study having enrolled 143 patients; 113 of them provided peri-ulcerative ileal biopsies and their associated stool DNAs. Conclusive results were obtained on 106/113 biopsies. Relevant clinical information such as HBI, calprotectin, CRP, and endoscopic scores were monitored.

The protocols for CrohnOmeter, PREDICT, and MOBIDIC studies were approved by the institutional medical ethics committees. Participants were given oral and written information prior to signing the informed consent form.

The HV cohort comes from the MICROLEAN study (Enterome sponsored with INRA MetaGenoPolis, Jouy-en-Josas, France and Universitätsspital Basel, Switzerland). Forty-three HV were enrolled, and no formal clinical information were collected. Each subject provided one stool sample collected at home.

### Stool sample preparation and processing for sequencing

Stool samples were collected at home using Sarstedt tubes (Sarstedt, Nümbrect, Germany) filled with RNAlater. On reception, the tubes were stored at – 80 °C. Sample processing, including DNA isolation, library preparation, and shotgun sequencing were performed by GATC Biotech (Konstanz, Germany). A commercial extraction kit, the QIAamp Stool DNA mini kit (Qiagen, Hilden, Germany) was used after optimization by GATC. DNA concentrations were measured using Qubit fluorometric quantitation (Life Technologies, Carlsbad, CA, USA). DNA libraries were prepared following the manufacturer’s instruction (Illumina, San Diego, CA, USA) and sequenced on a HiSeq 2500 Illumina sequencer. The target of 40 million minimum paired-end reads was generated for each sample, and sequencing read length was 100 to 125 bp.

### Bioinformatics processing for metagenomic stool samples sequencing

The resulting FASTQ files were processed using a customized version of the MOCAT pipeline [38]. Quality trimmed and filtered reads (PHRED quality cut-off 20) were then mapped against Enterome’s proprietary CD catalog including 4 million genes identified from the microbiome of healthy individuals and patients with CD. Sequence reads shorter than 45 bp, mapping to Illumina adapters, or to the human genome (GRCh37) were discarded. Bacterial genes were quantified using relative abundance measurements. Gene abundances for each sample were estimated as the sum of uniquely mapped reads per gene divided by the gene length and scaled by the sum of all the reads mapped on the microbiome gene catalogue [39]. The microbiome gene catalogue construction and annotation method were adapted from Li et al. [40]. Genes were annotated using the BLASTN alignment method against KEGG and RefSeq genomic databases [41] with different identity cut-offs for gene annotation at the species (95%), genus (80%), and phylum (65%) levels, requiring at least 80% of query sequence coverage. Genes with multiple hits deprived of any consensus (defined as 10% of hits having the same annotation) for their taxonomic associations were discarded. The relative abundances at each taxonomical level were computed by summing the relative abundances of all the genes belonging to the same species, family, genus or phylum. Species annotated to “others” and species annotated to “Homo Sapiens” were excluded from the analysis.

### Quantitative PCR for *FimS *ON and *FimS* OFF

DNA was extracted from 1 g of stool using the QiAmp DNA stool kit (Qiagen, Hilden, Germany). Gene sequences were obtained from an internal database and LF82 sequencing and were used to design q-PCR primers for *FimS* ON (forward primer: CGGATTATGGGAAAGAAAT; reverse primer: CGATGCTTTCCTCTATGA) and *FimS* OFF (forward primer: CGATGCTTTCCTCTATGA; reverse primer: TTGTTTTGTCAACGAGTT). The q-PCR was performed with 1X SYBR Green master mix in a 10-μl reaction volume. The reactions were carried out in 96-well plates. Each reaction comprised 2 μl of DNA, 0.4 μl of primer (10 μM each), 2.6 µl of RNase free water, and 5 μl of 2X SYBR Green master mix. The initial denaturation time was 1 min at 95 °C, followed by 45 cycles at 95 °C for 5 s, 60 °C for 30 s (annealing), 55 °C for 30 s (elongation), 72 °C for 10 s (extension). Following PCR amplification, a dissociation curve was run to examine the amplification specificity. A portion of the DNA was diluted and was used for primer validation and determination of optimal template dilutions.

### Cladogram representation

The cladogram (Supp Fig2) was built using the ggtree package [42]. We extracted from Ensembl database the list of species available in the human gut microbiome and those having a *FimH* gene. The clustering was done using 16S genes for each of these bacteria. The tree was built using MUSCLE software with its default parameters [43].

### Isolated bacterial strains

A total of 105 isolated *E. coli* from intestinal mucosa and fecal samples were collected from Crohn’s disease patients at the Laboratory of Molecular Microbiology, Biology Department, Universitat de Girona, Girona, Spain [7] and College of Veterinary Medicine, Cornell University, Ithaca, NY, USA [6] (Supplementary Table 4).

### DNA isolation of bacterial strains and full genome sequencing

DNA isolation for whole genome sequencing of bacterial strains was outsourced to GATC Biotech (Konstanz, Germany). A commercial extraction kit, the QIAamp Stool DNA mini kit (Qiagen, Hilden, Germany) was used to obtain at least 500 ng of DNA, as required for the library preparation. To control for contamination during DNA extraction, either a Tris–HCL buffer as negative control or an aliquot of the culturing media was used. The Qubit assay (Life Technologies, Carlsbad, CA, USA) was used to assess the DNA yield and purity using Abs_260/280 nm_. Fragmentation using gels was also performed on bacterial DNA to ensure that high molecular weight DNA was present. The genome sequencing was carried out as 300 bp paired-end with a MiSeq, using the V4 chemistry from Illumina, which did not vary for the duration of this project to ensure continuity. Negative controls were added to each of the sequencing runs, and a final quality control took place. A PhiX control was used to control the sequencing process.

### Bioinformatics processing for *E. coli* sequencing data

The *Escherichia coli* strain LF82 sequence (BioSample SAMEA3138414 on NCBI) was downloaded from NCBI and was used as a reference for the mapping of the 105 sequences from the *E. coli* isolates. This strain contains 4 376 genes. Alignment of the LF82 genes with the 105 isolated *E. coli* genomes was done using 95% of identity and 90% of coverage as criteria of mapping using BLASTN.

### TAK-018 stock solution preparation

Stock solutions of TAK-018 were prepared in dimethyl sulfoxide (DMSO) at a concentration of 10 mM and stored at – 20 °C until use. The TAK-018 concentrations tested were prepared in pure DMSO by tenfold serial dilution from the 10 mM stock solutions. Final DMSO concentration was 0.1%. Intermediate solutions were extemporaneously diluted in Ham’s F12 + Dulbecco’s modified Eagle’s medium (DMEM) (1:1) + 2 mM Glutamine + 10% Fetal bovine serum (FBS) + 1X Penicillin/streptomycin (used for T84 cell adhesion assays) and Luria–Bertani (LB) broth for the aggregation tests.

### In vitro testing of TAK-018 in prevention of bacterial adhesion on T84 epithelial cells

#### T84 cell line culture

The T84 human carcinoma cell line (Sigma Aldrich) was cultured in Ham’s F12 + DMEM (1:1) + 2 mM Glutamine + 10% Fetal bovine serum (FBS) + 1X Penicillin/streptomycin. T84 cell lines were not used between 9 and 15 passages. Four days before infection, 40,000 cells/well were seeded on black 96-well plates coated with poly-D-lysine. Medium (without antibiotics) was changed every 2 days, and cells were grown until confluence.

#### Bacterial adhesion assay

The media from the confluent 96-well plate was discarded, and 50 μl of compound solutions (0.1 nM, 1 nM, 10 nM, and 100 nM), compound vehicle, or media were added. Bacterial adhesion was tested using 50 μl of AIEC for 40 min (LF82; multiplicity of infection (MOI) of 10) or 70 min (NRG857c; MOI of 100) incubation periods. The plate was centrifuged at 1200 rpm for 5 min before incubation at 37 °C, 5% CO_2_. Supernatants were removed, and the plate was washed to remove non-adherent bacteria and other debris. Cells were then fixed with 4% paraformaldehyde (PFA), incubated at room temperature (RT) for 30 min and washed with phosphate buffered saline (PBS). The plate was kept at 4 °C until immunostaining. PBS was discarded, cells were permeabilized using 0.2% triton in PBS, incubated 15 min at RT, washed before addition of 1:200 of goat anti-*E. coli* (anti-O and anti-K antigens) and incubated for a further 2 h at RT. Then, cells were washed again and incubated with 1:200 donkey anti-goat Alexa 488. Cells were washed, and 1:2 4’,6-diamidino-2-phenylindole (DAPI) was added, plates incubated 5 min at RT in the dark. Image acquisition was performed by high content imaging (HCI) technology. This methodology allows to visualize and quantify the bacterial adhesion to T84 cells and assess the TAK-018 efficacy. To study the ability of TAK-018 to dislodge bound AIEC, adhesion of bacteria to T84 cells was first run 45 min and, subsequently, unbound bacteria were washed. Increasing concentration (as above) of TAK-018 was added and plates incubated for 45 min. Thereafter, cells were washed again and subjected to the same procedure used in the adhesion assay to quantify bacteria.

### Aggregation assay

The diverse bacterial suspensions (50 μl) were dispensed into 96-well plates, and 50 μl of the TAK-018 solutions (1 nM, 10 nM, 100 nM, 1 μM, 10 μM, and 100 μM) or LB with 0.2% DMSO were added. Plates were incubated for 5 h at 37 °C under slow agitation (200 rpm). Every hour, each well was observed with a microscope and the onset of aggregation was recorded. Bright field images were acquired to assess bacterial aggregation after 5 h of incubation.

No growth is indicative of samples for which, upon overnight culture, no bacterial growth could be observed. Therefore, no aggregation test could be performed. Negative is indicative of patients for which harvested bacteria grew but did not aggregate in the presence of TAK-018, indicative potentially of the lack of FimH expression.

### Measurement of transepithelial electric resistance (TEER)

T84 cells at 80–90% of confluency were detached from 25 cm^2^ flasks in 2 ml of trypsin-ethylenediaminetetraacetic acid (EDTA) solution. After centrifugation (1200 rpm, 5 min), cells were resuspended in culture medium, cells were counted using Malassez’s cell, and the cell density was adjusted to approximately 200,000 cells/ml by dilution in culture medium. Cells were then plated onto 12-well inserts (ThinCert Greiner, diameter 1 cm^2^, pore size 3 µm, ref 665,630 Dominique Dutscher) at ~ 100,000 cells per well (500 µl of cell suspension). Cells were left to attach and differentiate for 21 days before bacterial infection with medium changes every 2–3 days. The day before the assay, tubes containing 3 ml of LB (from Sigma Aldrich, reference: L3522) were inoculated with glycerol stock of bacteria maintained at − 8 0 °C using sterile loops. Tubes were incubated overnight at 37 °C without shaking. The next day, optical density of the bacterial suspensions was read at 600 nm allowing estimation of the bacterial cell density. Bacterial suspensions were centrifuged at 3000 rpm, 10 min at 4 °C. Bacterial pellets were resuspended in DMEM without antibiotic or serum, and bacterial concentrations were adjusted at 10^9^ bacteria/ml. Bacteria were then incubated 1 h at 37 °C with TAK-018 at three final concentrations (1 nM, 100 nM, 1 µM) or the vehicle DMSO (0.1% final concentration). In parallel, inserts of T84 cells were aspirated, and cells were washed 3 times with DMEM without antibiotic or serum. T84 cells were then exposed to 500 µl of bacteria suspensions exposed or not to TAK-018 or DMSO. Cells were also exposed to LPS or Flagellin pre-incubated or not with DMSO or TAK-018 at 1 nM, 100 nM, and 1 µM. LPS was from *E. coli* K12 (from Invivogen, ref: tlrl-eklps) and was used at 100 ng/ml as positive control of hTLR4 activation. Flagellin was from *Salmonella typhimurium* (from Invivogen, ref: tlrl-epstfla-5) and was used at 10 µg/ml as agonist of TLR5 receptor. Transepithelial electrical resistance (TEER) was measured before infection (at t0) using Millicell-ERS (electrical resistance system) Voltohmmeter (Millipore, ref: MERS00002). After 4 h at 37 °C, TEER was again measured, and basolateral compartments were collected and stored at – 80 °C before measurement of cytokine levels using enzyme-linked immunosorbent assay (ELISA) kits (detection kits for human IL-1β, IL-6, IL-8, or TNF-α; BD Biosciences ref: 557953, 555220, 555244, 555212, respectively).

### In vitro experiments using HEK-TLR4^+/+^ and HEK-TLR4^−/−^ cells

#### HEK cell culture

HEK-TLR4^−/−^ (reference: hkb-null2) and HEK-hTLR4 (reference: hkb-hTLR4) cells were obtained from Invivogen and grown accordingly to manufacturer’s instructions. HEK-TLR4^−/−^ cells were grown in the following medium: DMEM, 4.5 g/l glucose, 2–4 mM L-glutamine, 10% (*v*/*v*) fetal bovine serum, 50 U/ml penicillin, 50 μg/ml streptomycin, 100 μg/ml Normocin, and 100 μg/ml of the selection antibiotic Zeocin. HEK-hTLR4 cells were grown in the following medium: DMEM, 4.5 g/l glucose, 2–4 mM L-glutamine, 10% (*v*/*v*) fetal bovine serum, 50 U/ml penicillin, 50 μg/ml streptomycin, 100 μg/ml Normocin, and HEK-hTLR4 selection antibiotics diluted 1:250 (stock at 250X, reference hb-sel).

#### Cytokine secretion assay

HEK-TLR4^−/−^ and HEK-hTLR4 cells at 80–90% of confluency were detached from 25 cm^2^ flasks in 2 ml of PBS using mechanical detachment technic with cell scraper (avoiding trypsin used accordingly to manufacturer’s recommendations). Cells were counted using Malassez’s cell, and the cell density was adjusted to approximately ~ 280,000 and ~ 140,000 cells/ml by dilution in culture medium. Cells were then seeded into 12-well plates (1 ml per well) and left to attach for 24 h before treatment. Cells were either left untreated or treated for 16 h at 37 °C with (i) increasing concentrations of purified E coli K12 FimH and (ii) LPS from E coli K12 (from Invivogen, reference: tlrl-eklps) used at 100 ng/ml as positive control of hTLR4 activation. After incubation, cell culture supernatants were collected and stored at – 80 °C before measurement of TNFa levels using ELISA kits (detection kits for human TNF alpha from BD Biosciences reference 555212).

### In vitro experiments using human primary enterocytes

#### Primary enterocyte cell culture

Primary intestinal cells were isolated from a surgical piece of a patient with CD, by digestion of the mucosae using proteases. Briefly, mucosae were stripped from intestinal resection and incubated in lactated Ringer’s (LR) buffer containing dithiothreitol (DTT) to remove mucus. The mucosae was then transferred to new LR buffer containing collagenase and pronase and homogenized by pipetting. Cell suspension was filtered through sterile gauze and centrifuged. Pellet was resuspended in DMEM containing 10% FBS (referred as complete media) and antibiotics (penicillin–streptomycin solution and gentamycin at 50 µg/ml). Cells were centrifuged and resuspended in complete media to obtain a cell density of 200,000 cells/ml. Cells were seeded into 96-well plates at 20,000 cells per well and incubated at 37 °C for 16–24 h before adhesion assay.

#### Adhesion assay

After incubation, wells were aspirated and washed with DMEM without serum and without antibiotics. Bacteria were pre-incubated for 1 h at 37 °C with increasing concentration of TAK-018 or with vehicle (DMSO, 1:1000 dilution). Then, primary cells were exposed to 10^9^ of AIEC and non-AIEC bacteria, exposed or not to TAK-018. In parallel to the bacteria, control wells were exposed to flagellin isolated from *Salmonella*, LPS from *E. coli* or human recombinant IL-1β for 4 h at 37 °C. Supernatant was collected and frozen at − 80 °C until determination of cytokines by ELISA. Cells were washed with PBS containing calcium and magnesium (PBS^++^) and fixed with 4% PFA diluted in PBS^++^ (100 µl per well). Wells were washed with PBS^++^ before determination of bacterial adhesion using ELISA assay.

#### Quantification of bacterial adhesion using ELISA assay

Primary antibodies directed against specific bacteria were incubated at 1:1000 in saturation buffer (PBS^++^ supplemented with 10% FBS). Primary antibodies used were *Mycobacterium avium* monoclonal antibody (ref. MA1-10788), *Klebsiella pneumoniae* rabbit polyclonal antibody (ref. PA1-7226), *Salmonella* rabbit polyclonal antibody (ref. PA1-7244), and *E. coli* serotypes O + K rabbit antibody (ref. PA1-7213) from ThermoFisher. After 1-h incubation at RT, wells were washed with PBS. Secondary antibodies goat anti-rabbit IgG (ref. 111-035-003) and goat anti-mouse IgG (ref. 115-035-003) from Jackson ImmunoResearch, diluted at 1:10,000 in saturation buffer and conjugated to horseradish peroxidase (HRP), were added (100 µl) and incubated for 1 h at RT. Wells were then washed with PBS, and HRP substrate (Sigma Fast-OPD, ref P9187) was added to reveal. Plates were incubated in the dark, before H_2_SO_4_ 2 N was added and optical density was measured at 490 nm.

### Ex vivo experiments on explants from patients with CD

#### Total adhesion measurements

Ex vivo experiments were performed on ileocecal resections from patients undergoing surgery and having agreed to use their tissues for research purposes. Only freshly prepared intestinal punches were used. TAK-018 compound was tested at 3 concentration levels on the adhesion of LF82-Green Fluorescent Protein (GFP) bacteria (10^9^/ml, 4 h of incubation) for a total of 4 conditions. Human recombinant IL-1β (final concentration 2 ng/ml) was used as positive control of pro-inflammatory signal. At the end of the 4-h incubation period with LF82-GFP bacteria, cell culture media was collected for cytokine quantification. After 6 washes with PBS (1 ml), explants were transferred to matrix lysis tubes and subjected to mechanical lysis using bead-better apparatus. Green fluorescence was quantified using black 96-well plates and a spectrofluorometric plate reader.

#### Determination of inflammation marker by ELISA

Supernatant (SN) of LF82-GFP and/or TAK-018–treated explants were collected after 4 h and stored at – 80 °C until cytokine determination. Cytokine levels in tissues (“Tissue” samples) were also measured after mechanical lysis of the explants in 500 µl of PBS containing protease inhibitors (Sigma Aldrich; ref. P8340). For cytokine determination, commercial ELISA kits, allowing detection of human IL-1β, IL-6, IL-8, or TNF-α, were used according to manufacturer’s instructions (BD Biosciences kits; refs: 557953, 555220, 555244, 555212, respectively).

#### Microscopy

For microscopy analysis, at the end of the 4-h incubation, the punches of tissue used were fixed in 1 ml of PFA 4% for 24 h. Punches were washed twice with 1 ml of PBS and cut in by half and included in inclusion medium (TFM), in transverse position to allow sectioning in the crypt–villosity axis. Then, 3 sections of 5-µm thickness were obtained per piece of punch, each section being separated by 60 µm from the next to cover all the tissue. Sections were stained with Phalloidin, conjugated to Alexa-547 to microscopically assess bacterial adhesion or stained with hematoxylin and eosin (H&E) to analyze histopathology damage.

### Statistical analysis

For the Cohort 1 from the CrohnOmeter study, data studied corresponds to the first timepoint available for each patient. For both cohorts of patients with CD, patients were considered “Active CD patients” if their HBI was > 4, “Quiet CD patients” otherwise. Clinical variables were summarized as medians with interquartile ranges (IQRs) or as frequencies with percentages. For non-paired samples, the nonparametric Mann–Whitney U test was performed to compare continuous variables between 2 groups and the nonparametric Kruskal–Wallis test was performed to compare continuous variables between more than 2 groups. For paired samples, nonparametric Wilcoxon’s signed-rank test for paired samples was performed to compare continuous variables between 2 groups/timepoints, and linear mixed models were performed to compare continuous variables between more than 2 groups/timepoints. Additionally, we studied only species detected (relative abundance > 0) in at least 70% of samples from at least one cohort. Additionally, for all metagenomics data analyses except the gene diversity, a pseudo-count of 1e-10 was added. ANOVA was performed for each group on the aggregation data from Fig. 4a and b. A *P* value lower than 0.05 was considered statistically significant. All *P* values presented come from two-sided tests. All statistical representations were added manually on figures. All statistical analyses were performed using R software (version 3.6.1).

### Data availability

The datasets analyzed during the current study are available (“Blockage of bacterial FimH prevents mucosal inflammation associated with Crohn’s disease”, Mendeley Data, V1, https://doi.org/10.17632/4s3f4dv59g.1). The informed consent does not allow us to share on a public domain the metagenomic data. However, these data are available from the corresponding author on reasonable request.

## Results

### Characteristics of study participants

We assessed the intestinal microbial communities of 401 individuals, including 358 patients with CD (from two distinct cohorts) and 43 healthy volunteers (HV), using shotgun metagenomic sequencing in fecal samples. All samples collected were analyzed using shotgun sequencing, and reads corresponding to different bacterial species were quantified and normalized relative to the total stool DNA in order to generate unbiased quantification of the different bacterial DNA species studied. Demographic data and history of CD and disease activity for each of the studies are described in Supplementary Table 1. Demographic data for the HV is presented in Supplementary Table 2. The first cohort of patients with CD, from the CrohnOmeter study, was followed longitudinally, and we did not observe statistically significant differences in terms of HBI (Supplementary Fig1a) and *E. coli* relative abundance (Supplementary Fig1b) across the different time points. Therefore, we chose to focus our analysis on the data corresponding to the first time point available, in order to compare this cohort with the second cohort, from the PREDICT study.

### Patients with CD show altered microbiota profiles characterized by a blooming of *Enterobacteriaceae*

**Fig. 1:**
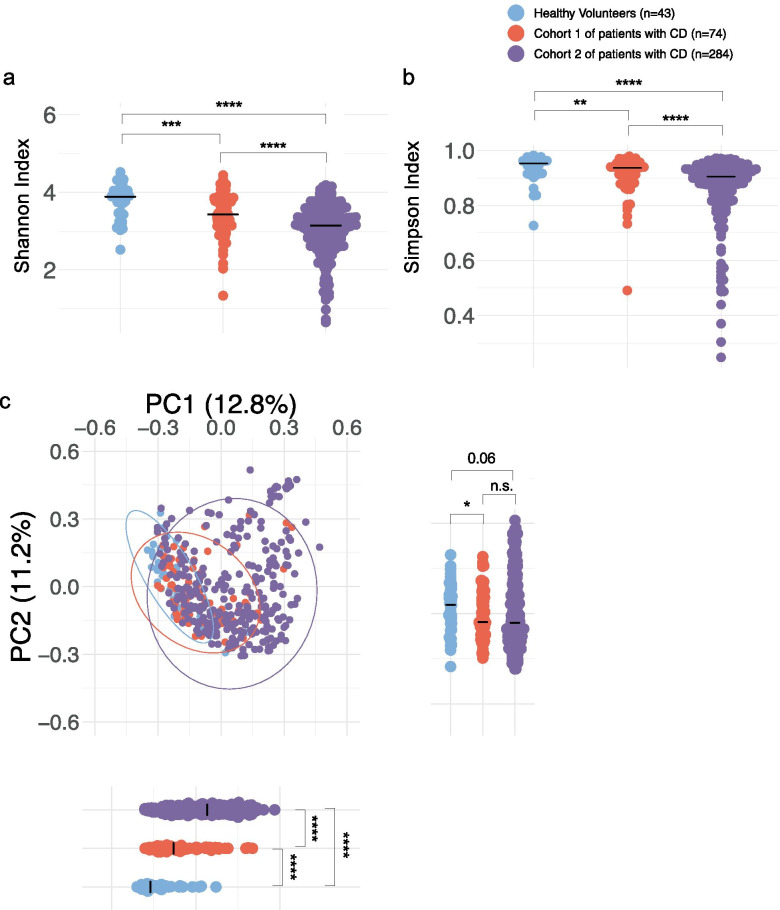
Altered microbiota profiles in patients with CD from 2 different cohorts. **a**, **b** Species diversity in patients with CD and healthy volunteers were calculated using alpha diversity: Shannon (**a**) and Simpson indices (**b**). **c** Microbial clustering is shown based on Bray–Curtis dissimilarity principal coordinate analysis (PCoA) metrics for Cohort 1, Cohort 2, and HV. Ellipsoids represent a 95% confidence interval surrounding each group. There were 74 patients with CD in Cohort 1, 284 patients with CD in Cohort 2, and 43 healthy volunteers. Bars represent the median of all points. Nonparametric Mann–Whitney U test was used to identify the statistically significant differences between groups. (*P < 0.05, **P < 0.005, ***P < 0.0005, ****P < 0.0001)

**Fig. 2:**
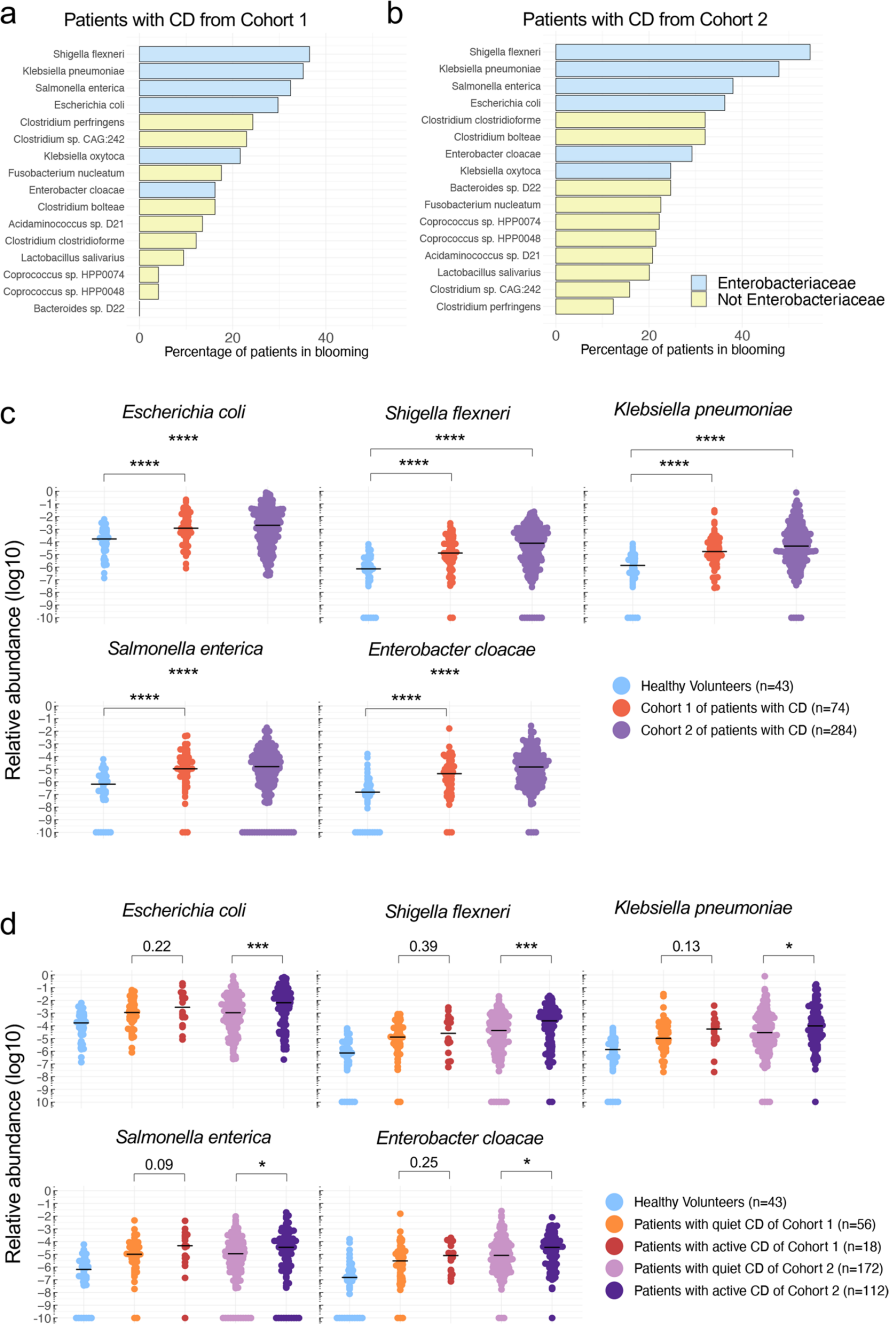
Fecal microbial composition of patients with CD is characterized by a blooming of *Enterobacteriaceae*. **a**, **b** Top blooming species by percentage of patients with CD in Cohort 1 (**a**) and Cohort 2 (**b**), for whom a tenfold increase has been observed compared with mean relative abundance in HV. Are represented only the species with at least 20% of patients in blooming either in Cohort 1 or in Cohort 2. Note the presence of 4 *Enterobacteriaceae* among the top 5 species. **c** Relative abundance of *Enterobacteriaceae spp* in HV and patients with CD from Cohort 1 and Cohort 2. **d** Relative abundance of *Enterobacteriaceae spp* in HV, active and quiet patients with CD from Cohort 1 and Cohort 2. Patients with CD were considered in an active phase of the disease when HBI > 4 and in a quiet phase when HBI ≤ 4. Bars represent the median of all points. Nonparametric Mann–Whitney U test was used to identify the statistically significant differences between groups. (*P < 0.05, ***P < 0.0005, ****P < 0.0001)

As expected, the overall alpha diversity, measured according to the Shannon and Simpson indices, was significantly reduced in samples from patients with CD when compared with those from HV, in both patient cohorts (Fig. 1a, b). The distinctiveness of the microbiota from patients with CD was confirmed by beta diversity analysis, demonstrating a clustering of samples according to CD diagnosis for both cohorts with the same homogeneity by Bray–Curtis dissimilarity metrics (Fig. 1c). We also observed significant differences between Cohort 1 and Cohort 2 regarding the Shannon and Simpson indices (Fig. 1a, b) as well as the Bray–Curtis dissimilarity metrics (Fig. 1c), likely because of differences in the inflammatory status of the patients between the two cohorts, illustrated by higher Harvey-Bradshaw index and higher calprotectin levels in cohort 2 (Supplementary Table 1). Determining the relative abundance of *E. coli* and other *Enterobacteriaceae spp*, we observed that *Enterobacteriaceae* were strongly enriched in patients with CD compared with HV: indeed, 30 to 55% of samples showed a greater-than tenfold increase in different *Enterobacteriaceae spp* including *Shigella flexneri*, *Klebsiella pneumoniae*, *Salmonella enterica*, and *Escherichia coli* (Fig. 2a, b and Supplementary Table 3), illustrating the blooming of *Enterobacteriaceae* observed in CD (species for which a tenfold increase has been observed in patients with CD compared with mean abundance in HV). Moreover, we observed a different clustering of the patients from Cohort 2 in the PC1 scores in the PCoA analysis (Fig. 1c). Indeed, some patient samples clustered with HV, but a larger fraction was very different from healthy controls (Supplementary Fig3a). This dichotomy of patient samples in Cohort 2 has been further investigated at the levels of *Enterobacteriaceae spp* abundances and we observed striking differences, with the population of patients from Cohort 2 overlapping HV on the PCoA having highly significantly lower *Enterobacteriaceae spp* abundance compared with the other patients from Cohort 2 (Supplementary Fig3b). In terms of relative abundances, *E. coli* species which in HV account for less than 0.02% of all fecal bacterial DNA, becomes a major species accounting for 1 up to 80% of all stool DNA in > 20% of all our samples from patients with CD. We next separated the samples according to the disease status of the donor (considered in an active phase of the disease when HBI > 4 and in a quiet phase when HBI ≤ 4) and observed an association between levels of *Enterobacteriaceae spp* and disease activity (Fig. 2c, d).

### FimH adhesin is expressed by bacteria in ileal biopsies from patients with CD

**Fig. 3:**
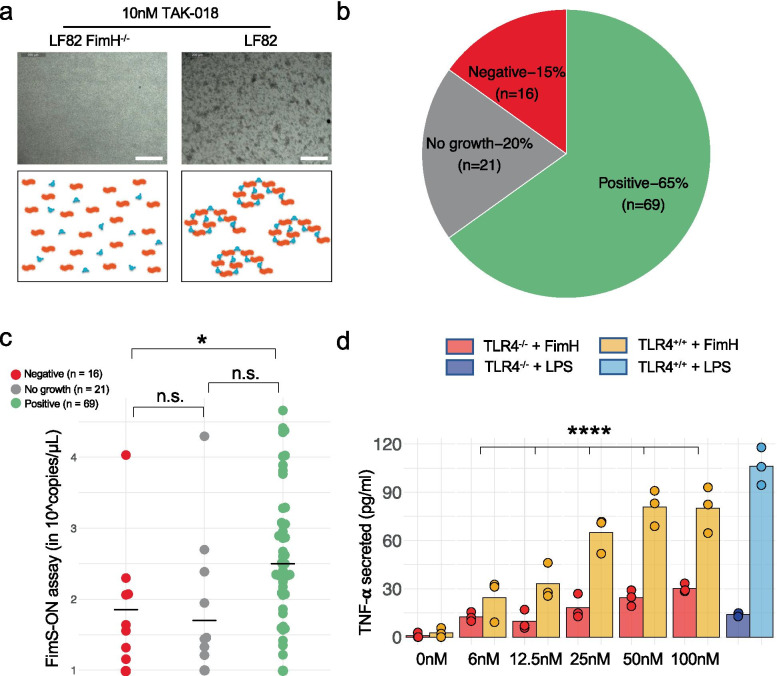
FimH adhesin is expressed by bacteria in ileal biopsies from patients with CD and induces inflammatory response in a TLR4-dependent manner. **a** Aggregation of LF82 *E. coli* upon incubation with TAK-018. Note that FimH^−/−^ LF82 *E. coli* did not aggregate upon incubation with *E. coli* illustrating that aggregation is a FimH-dependent phenomenon. **b** Presence of FimH-expressing bacteria in patients with CD as measured by aggregation of bacteria to TAK-018 in ileal biopsies. One to two biopsies were analyzed per patient. **c** FimS-ON expression in fecal samples from patients with CD, as measured by qPCR. Bars represent the median of all points. Nonparametric Mann–Whitney U test was used to identify the statistically significant differences between groups (*P < 0.05; *n.s*. nonsignificant). **d** Secretion of TNF-α of HEK-hTLR4^+/+^ and HEK-TLR4^−/−^ cells upon incubation with increased concentration of FimH. LPS was used as a positive control at a concentration of 100 ng/ml. FimH was produced in yeast in order to be LPS-free. The data are centered on the mean value at 0. Bars represent the mean of all points. An analysis of covariance (ANCOVA) was used to identify the statistically significant difference between TLR4^−/−^ and TLR4^+/+^ groups upon FimH treatment (****P < 0.0001; 0 nM and LPS conditions were excluded from the ANCOVA)

At the metagenomic level, we confirmed that FimH presence was restricted to a family of *Proteobacteria*, all species of the *Enterobacteriaceae* family expressing FimH (Supplementary Fig2) [18]. We also tested for the presence of FimH-expressing bacteria in ileal biopsies in a third cohort of patients with CD from the MOBIDIC study (Supplementary Table 1). In order to do so, we used our designed bi-mannosylated compound TAK-018 (EB8018/Sibofimloc) that sensitively identifies FimH expression [44, 45] (Supplementary Fig4). We showed that TAK-018 aggregates the reference FimH-expressing AIEC *E. coli* LF82 strain, whereas a FimH negative mutant of *E. coli* LF82 did not aggregate upon incubation with TAK-018, allowing for the detection of FimH-expressing bacteria with TAK-018 (Fig. 3a). We observed that 65% of patients (69/106 patients) did display adherent colonies made up of FimH-expressing bacteria, as measured by aggregation to TAK-018 (Fig. 3b). Moreover, we characterized the relationship between FimH and the “AIEC” phenotype by sequencing a total of 98 isolated *E. coli* strains described as “AIECs” (Supplementary Table 4). We correlated sequencing data with results from the aggregation assay and mapped sequence reads against the *E. coli* LF82 strain used as reference, and specifically focused on the *fim* operon. The mapping against the *fimS* region revealed a strong association between the percentage of reads in the “ON” position, indicative of expression of the entire *fim* operon, and the aggregation to TAK-018 [46] (Supplementary Fig5). When applied to the samples from patients with CD, we also observed a strong relationship between the level of *fimS*-ON analyzed by qPCR in feces and functional expression of FimH in ileal biopsies from these samples (Fig. 3c).

### FimH blocker TAK-018 prevents *Enterobacteriaceae* adhesion and inflammation in gut explant in a TLR4-dependent manner

FimH has been described as a potent TLR4 agonist in the gut which can trigger inflammation independently of the presence of lipopolysaccharides (LPS) [16], even though TLR4 expression is upregulated in ileal biopsies from patients with CD [23, 28, 47]. As previously reported [15–17], we confirmed a dose-dependent increased secretion of tumor necrosis factor alpha (TNFα) by epithelial human embryonic kidney (HEK) cells upon incubation with purified FimH, which was completely abrogated in TLR4^−/−^ HEK cells (Fig. 3d). Considering that TLR4 expression is upregulated by inflammatory cytokines in ileal biopsies from patients with CD [23, 28, 47] and that an inflamed environment is conducive to blooms of *Enterobacteriaceae* [48], we hypothesized a critical role for FimH as a pro-inflammatory trigger leading to a vicious circle in CD, stressing the importance of designing therapeutic strategies that can disrupt this pathogenic loop.

**Fig. 4:**
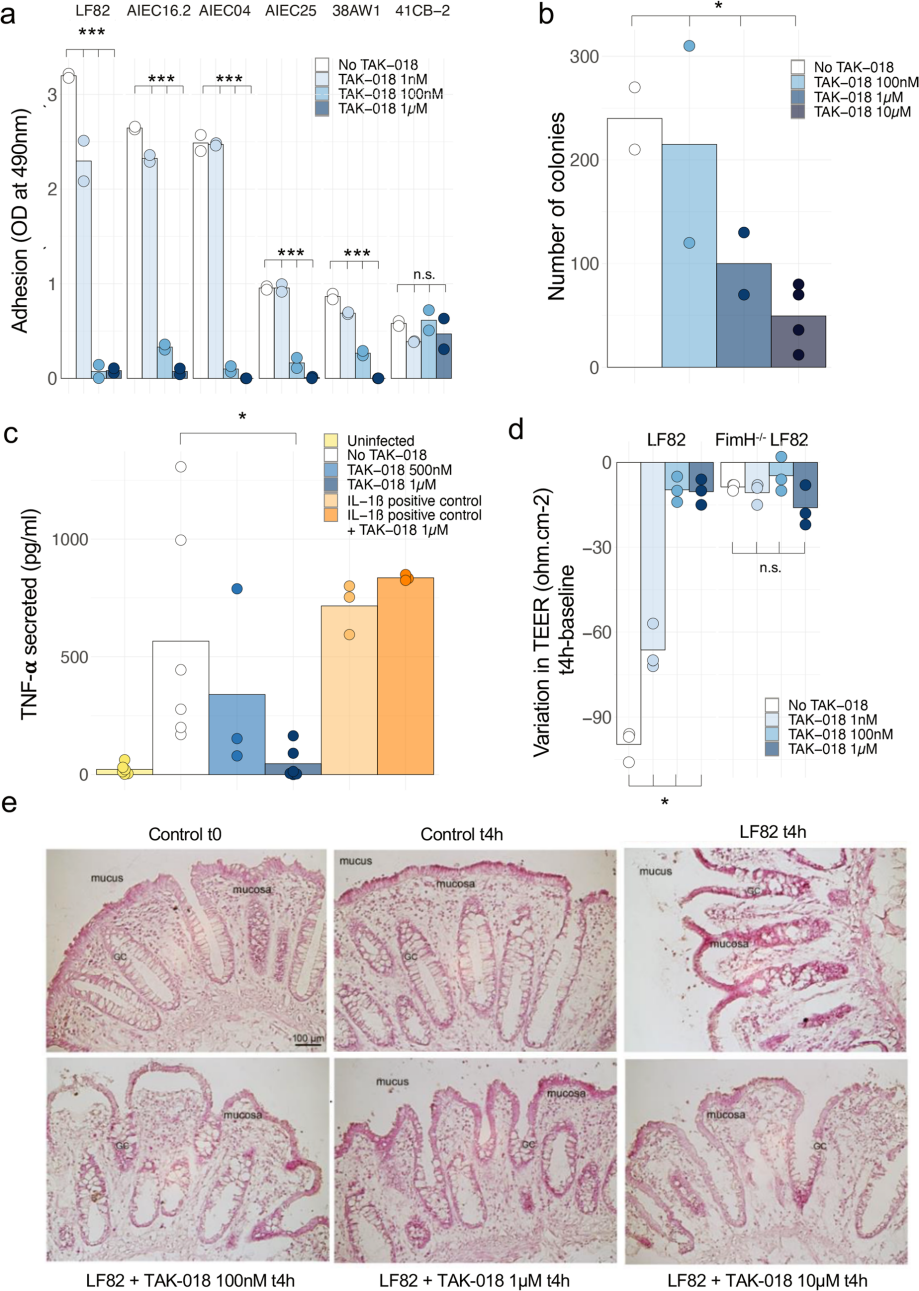
FimH blocker TAK-018 prevents *Enterobacteriaceae* adhesion and inflammation of gut explant. **a** Adhesion of different strains of *E. coli* to human primary intestinal epithelial cells, in the presence of increasing concentrations of TAK-018. Note that the 41CB2 strain did not express FimH. Bars represent the mean value of individual points which themselves correspond to biological replicates. ANOVA was performed for each group to identify the statistically significant effect of TAK-018 (***P < 0.001). **b** Number of intracellular colonies in epithelial cells from human ileal explant incubated with LF82 *E. coli*, in the presence of increasing concentrations of TAK-018. Bars represent the mean value of individual points which themselves correspond to biological replicates. ANOVA was performed to identify the statistically significant effect of TAK-018 (*P < 0.05). **c** TNF-α secretion of human ileal explant incubated for 4 h with 10^9^ LF82 *E. coli*, in the presence of increasing concentrations of TAK-018. IL-1β was used as a positive control to trigger inflammation. Bars represent the mean value of individual points which themselves correspond to biological replicates. A nonparametric Wilcoxon signed-rank paired test was used to identify the statistically significant differences between the LF82 and LF82 + TAK-018 1 µM groups (*P* = 0.031). A linear mixed model was used to identify the statistically significant differences between the groups LF82, LF82 + 500 nM, and LF82 + 1 µM (*P* = 0.07). **d** Variation of transepithelial electric resistance (TEER) of T84 cells incubated for 4 h with 10^9^ LF82 *E. coli*, in the presence of increasing concentrations of TAK-018. Note that FimH^−/−^ LF82 *E. coli* does not adhere to T84 cells and therefore does not affect TEER. Bars represent the mean value of individual points which themselves correspond to biological replicates. A nonparametric Kruskal–Wallis test was used to identify the statistically significant differences between the LF82 groups (*P* = 0.02). **e** Histopathologic effects of LF82 *E. coli* adhesion on human ileal explants from a patient with CD and respective countereffects in the presence of increasing concentrations of TAK-018. Note the characteristic mucosa desquamation upon 4-h incubation with LF82 *E. coli*, visibly reduced in the presence of increased concentrations of TAK-018

Accordingly, we studied the effects of blocking bacterial adhesin FimH with TAK-018 by performing adhesion assays on AIEC and non-AIEC strains to epithelial cells. We observed that different bacteria described as AIECs (most notably *E. coli* LF82) are able to adhere to human primary intestinal cells in a FimH-dependent manner, except the 41CB2 strain that does not express FimH (Fig. 4a and Supplementary Table 4). Moreover, we demonstrated a concentration-dependent decrease in bacterial adhesion by TAK-018 with full blocking activity observed at 1 μM TAK-018 for all strains (Fig. 4a), also confirmed on T84 epithelial cell line (Supplementary Fig6) and primary ileal cells (Supplementary Fig7). This blocking of bacterial adhesion further prevented intracellular infection of epithelial cells by LF82 *E. coli* (Fig. 4b).

We also determined the effect of preventing FimH sensing by TLR4 with TAK-018 on cytokine secretion. *E. coli* LF82 induced a robust secretion of TNFα, IL-6, and IL-8, TAK-018 inhibiting dose-dependently the secretion of all three cytokines, with 1 μM retaining levels close to or below basal ones (Fig. 4c and Supplementary Fig8). We further assessed the functional implications in terms of tight junction dynamics and gut permeability by determining transepithelial electrical resistance (TEER) in T84 cells. *E. coli* LF82 induced a robust decrease in TEER in a FimH-dependent manner; as a FimH-deleted LF82 mutant did not affect TEER [49]. These effects were dose-dependently counteracted by TAK-018, with full efficacy at 1 µM (Fig. 4d). Similarly, histological analyses of the human gut explants incubated with *E. coli* LF82 showed an extremely pronounced inflammatory effect on the intestinal tissue with mucosa desquamation (Fig. 4e). Incubation with increasing amount of TAK-018 showed again gradual decrease in the inflammatory reaction and a general and clear improvement of gut explant integrity, with a concentration of 1 μM TAK-018 being able to maintain normal tissue integrity (Fig. 4e).

## Discussion

The present study documents and quantifies an enrichment of *E. coli* and other *Enterobacteriaceae spp* in the gut of patients with CD. Our data suggest that a blooming of FimH-expressing bacteria could participate in initiating inflammation patients with CD. We observed potentially pathogenic levels of *Enterobacteriaceae* in over 75% of patients with CD when followed longitudinally. On human ileal resections, FimH contributes to mucus colonization and decreased mucus thickness, epithelial adherence and inflammation, and desquamation. Moreover, in animal models of colitis, FimH has been shown to be required to induce severe inflammatory damages since genetic deletion of FimH led to less engraftment and inflammation upon gavage with FimH-mutant LF82 AIEC [49]. The pathogenesis of AIECs has been linked to the overexpression of the *fim* operon [50]. The clinical cohorts reported here show an increased number of FimH-expressing bacteria associated with CD and with disease activity that account for the top 4 bloomers. The gene *fimH* is under strict control of a “switch” mechanism of the invertible *fimS* promoter [34, 51]. A qPCR strategy was designed using primers that amplify the level of *fimS*-ON in stool samples. The results showed that *fimS*-ON in stools is statistically linked to aggregation in ileal biopsies, suggesting that this might be a reliable and noninvasive tool to diagnose patients with CD hosting FimH-expressing bacteria. However, we did not perform metagenomic analysis of fecal samples and cannot infer whether AIEC levels would correlate between fecal and ileal samples. To date, among human gut bacteria, only FimH proteins and in particular *E. coli* FimH proteins, are annotated with the gene ontology term “mannose binding” (GO:0005537) on the UniProt database. To our knowledge, there is no other gene in *Enterobacteriaceae* capable of recognizing mannose. These bacteria interact with TLR4 receptors, insensitive to LPS in the gut, through its FimH, inducing large release of TNFα [16]. The gene *fimH* is not constitutively expressed in non-inflammatory conditions, and therefore, these commensals are not harmful for the host. Once the biofilm is formed, FimH-expressing bacteria can invade the mucosa by transcytosis of the epithelial cells and are also phagocyted by M cells in Peyer’s patches [52].

In the current status of drug-resistant bacterial pathogens, the availability of antibiotics to effectively treat patients is limited and can even further aggravate dysbiosis by killing the beneficial bacteria and allow overgrowth of the resistant species. Traditional treatments and novel therapies for CD include biologic agents that target various mechanisms of action in the inflammatory pathways [53], but they do not target the source of the inflammation that originates from an imbalanced gut microbiota and the emergence of pro-inflammatory pathogenic bacteria. Elimination of pathogenic FimH-carrying bacteria from the gut would thus represent a selective strategy to suppress a potentially critical trigger of intestinal inflammation.

We have shown using the AIEC reference strain LF82 expressing FimH that targeting FimH could prevent bacterial adhesion and alleviate inflammation in ileal explants. Considering that all members of the *Enterobacteriaceae* family express FimH [18], we assume that this strategy would also be relevant for other *Enterobacteriaceae* species. However, we did not test our FimH blocker in animal models and chose to move forward into the clinic, based on these results. Therefore, even though our data suggest that FimH is an inflammatory trigger in CD, the initiation of a phase Ib clinical study in patients with CD (NCT03709628) will bring the expected information whether FimH blockage represent a realistic strategy for treating patients with CD. Targeting the bacterial adhesion of FimH-expressing bacteria is a promising therapeutic method that consists of ‘disarming’ bacteria without killing them, representing a selective strategy to suppress a potentially critical trigger of intestinal inflammation, without disturbing the intestinal microbiota [54–58]. In addition, this therapeutic approach exerts weaker selective pressure compared with other antibacterial agents and thus the emergence of bacterial resistance is expected to be residual. Accordingly, to interfere with AIEC adhesion to host cells, the development of drugs rationally designed to saturate the carbohydrate recognition domain (CRD) of FimH, by mimicking its natural ligand, appears of great interest for a better personalized therapeutic strategy to manage CD [35].

## Conclusions

Considering the limits of other therapies, TAK-018 has been designed as a novel, first-in-class small molecule FimH blocker, orally administered and gut-restricted with minimal systemic absorption [58]. We have shown here that the microbiome of patients with CD was enriched with *Enterobacteriaceae spp,* this enrichment being even more pronounced in patients with active CD. As *Enterobacteriaceae* express the bacterial adhesin FimH, we confirmed the presence of bacterial FimH expression in ileal biopsies from patients with CD, suggesting a pathophysiological role for FimH-expressing bacteria in CD. Using human intestinal explants, we further showed that FimH is essential for adhesion and to trigger inflammation. Finally, a specific FimH blocker, TAK-018, was shown to reduce bacterial adhesion in a dose-dependent manner, totally blunting inflammation and preserving tissue integrity. Moreover, we have conducted a 2-week phase Ia clinical study where TAK-018 has been shown to be well tolerated in healthy volunteers with a maximum daily dose of 3000 mg/day (NCT02998190) [58]. The benefit–risk balance of TAK-018 enabled the initiation of a phase Ib clinical study in patients with CD to assess the pharmacokinetics, under TAK-018 designation (NCT03709628) [59]. Because of its properties and mode of action, TAK-018 can be especially useful in maintenance therapy and be complementary to other more symptomatic therapeutics. Accordingly, TAK-018 is currently evaluated in postoperative CD in a phase II study.

## Supplementary Information

**Additional file 1: Supplementary Table 1.** Demographic data and history of Crohn’s Disease of the patients with CD included in each of the presented studies. *: Montreal classification for Cohort 1 and 3. **: MOBIDIC: QUANTA Lite/LLOD=15.6mg/kg. CrohnOmeter: Bühlmann/LLOD=50mg/kg. PREDICT: Bühlmann/LLOD=30mg/kg. NA=Not available. **Supplementary Table 2.** Demographic data of the healthy volunteers included in the presented study. **Supplementary Table 3.** Median values for *Enterobacteriaceae* species in Fig. 2c (top table) and d (bottom table). **Supplementary Table 4.** List of isolated *E. coli* strains and associated information. **Supplementary Figure 1.** Evolution over time of the first cohort regarding HBI (left) and *E. coli* relative abundance (right). A linear mixed model was used to identify statistically significant differences between time points (visits) and did not reach significance for *E. coli *abundance (*P* = 0.51) nor for HBI (*P* = 0.41). **Supplementary Figure 2.** Some *Proteobacteria* express FimH adhesin. Cladogram representing the bacteria phyla detected in the human gut microbiome (left) and FimH presence with a focus on *Enterobacteriaceae spp *(right). **Supplementary Figure 3.** Dichotomy in patient with CD from Cohort 2. a, Microbial clustering as shown based on Bray–Curtis dissimilarity principal Coordinate Analysis (PCoA) metrics for HV and patients with CD from Cohort2 with PC1 ≤ 0.1 and PC1 > 0.1. Ellipsoids represent a 95% confidence interval surrounding each group. b, Relative abundance of *Enterobacteriaceae spp* in HV and patients with CD from Cohort2 with PC1 ≤ 0.1 and PC1 > 0.1. Non-parametric Mann-Whitney U test was used to identify the statistically significant differences between groups. (* *P* < 0.05, ** *P* < 0.005, **** *P* < 0.0001). **Supplementary Figure 4.** Structure of the bi-mannosylated FimH-blocker TAK-018. **Supplementary Figure 5.** Association between percentage of FimS-ON expression and aggregation to TAK-018 of different AIEC strains. The mapping against the *fimS* region revealed a strong association between the percentage of reads in the “ON” position, indicative of expression of the entire *fim* operon, and the aggregation to TAK-018. Median are represented. Non-parametric Mann-Whitney U test was used to identify the statistically significant differences between groups (n.s. non-significant, *** *P* < 0.0005, **** *P* < 0.0001). **Supplementary Figure 6.** TAK-018 prevents adhesion of LF82 *E. coli* to T84 intestinal epithelial cells in a FimH-dependent manner. Bars represent the mean value of individual points which themselves correspond to biological replicates. A non-parametric Kruskal-Wallis test was used to identify the statistically significant differences between the LF82 groups (*P* = 0.0002). **Supplementary Figure 7.** TAK-018 prevents pro-inflammatory cytokine secretion of human ileal explants upon incubation with LF82 *E. coli*. IL-6 and IL-8 secretion of human ileal explants incubated for 4 hours with 109 LF82 *E. coli*, in the presence of increasing concentrations of TAK-018. IL-1β was used as a positive control to trigger inflammation. Bars represent the mean value of individual points which themselves correspond to biological replicates. A linear mixed model was used to identify statistically significant differences between the groups No TAK-018, TAK-018 500nM and TAK-018 1µM (IL-6, *P* = 0.1; IL-8, *P* = 0.0006). **Supplementary Figure 8.** TAK-018 prevents adhesion of LF82 *E. coli* to primary human intestinal cells isolated from patient with Crohn’s disease. Microscopy images of GFP LF82* E. coli* (green) on primary human ileal cells stained with phalloidin Alexa-547 (red) in presence of TAK-018 (bottom) or not (top).

## Data Availability

The datasets analyzed during the current study are available (“Blockage of bacterial FimH prevents mucosal inflammation associated with Crohn’s disease”, Mendeley Data, V1, https://doi.org/10.17632/4s3f4dv59g.1). The informed consent does not allow us to share on a public domain the metagenomic data. However, these data are available from the corresponding author on reasonable request.
