# Supplementary material for: Blockage of bacterial FimH prevents mucosal inflammation associated with Crohn’s disease
Source: Microbiome. 2021 Aug 23;9:176. doi: 10.1186/s40168-021-01135-5 (PMC8383459; doi:10.1186/s40168-021-01135-5)

Supplementary Table 1

Study	Cohort 1: CrohnOmeter	Cohort 2: PREDICT	Cohort 3: MOBIDIC
**N**	98	284	143
**Age, median (years) (Q1; Q3)**	30.0<br>(25.0 ; 41.5)	39.50<br>(29.00 ; 51.00)	36.0<br>(28.0 ; 48.0)
**Sex (% female)**	71.4%	52.8%	55.9%
**BMI, median (kg/m^2) (Q1 ; Q3)**	21.52<br>(19.96 ; 24.46)	24.78<br>(22.09 ; 28.74)	25.11<br>(21.67 ; 27.14)
**Ethnicity**	NA	Caucasian – 93%<br>Black – 5.6%<br>Asian – 1.4%	Caucasian – 94.4%<br>Black – 4.9%<br>Asian – 0.7%
**Country**	EU - 100%	US + Canada – 76.1%<br>EU - 23.9%	US - 25.2%<br>EU - 74.8%
**Smoking status**	Current smoker – 25.6%<br>No smoker – 74.4%	Current smoker - 17.6%<br>No smoker - 82.4%	Current smoker - 25.2%<br>No smoker - 74.8%
**Age at diagnosis (years)**	≤16: 12.9%<br>[17- 40]: 83.5%<br>>40: 3.5%	≤16: 19.5%<br>[17- 40]: 62.1%<br>>40: 18.4%	≤16: 17.6%<br>[17- 40]: 65.5%<br>>40: 16.9%
**Current localization***	L1 – 38.0%<br>L2 – 5.6%<br>L3 – 56.3%<br>L4 – 7.0%	Ileum – 76.1%<br>Colon – 58.8%<br>Rectum – 14.1%<br>Anal – 9.9%	L1 – 37.3%<br>L3 – 62.7%
**Bowel resection (% population)**	NA	27.8%	49.7%
**CDAI, median (Q1 ; Q3)**	NA	130.3<br>(80.4 ; 195.8)	91.0<br>(45.4 ; 182.9)
**HBI, median (Q1 ; Q3)**	2.0<br>(0.3 ; 4.0)	3.0<br>(1.0 ; 7.0)	3.0<br>(1.0 ; 5.3)
**Calprotectin**, median (Q1 ; Q3) (mg/kg)**	71.9<br>(50.0 ; 280.9)	205.0<br>(64.0 ; 786.0)	35.8<br>(15.6 ; 164.5)

Supplementary Table 2

	Healthy volunteers
**N**	43
**Age, median (years) (Q1 ; Q3)**	38 (34.3 ; 43.8)
**Sex (% of female)**	63%
**BMI, median (Q1 ; Q3) (kg/m^2)**	22.6 (21.6 ; 24.4)
**Country**	EU - 100%

Supplementary Table 3

	HV	Cohort 1	Cohort 2
Escherichia coli	1,7E-04	1,2E-03	2,0E-03
Shigella flexneri	7,3E-07	1,3E-05	7,8E-05
Klebsiella pneumoniae	1,4E-06	1,8E-05	4,6E-05
Salmonella enterica	6,5E-07	1,1E-05	1,6E-05
Enterobacter cloacae	1,5E-07	4,4E-06	1,5E-05

	HV	Cohort 1		Cohort 2	
		Quiet	Active	Quiet	Active
Escherichia coli	1,7E-04	1,1E-03	4,0E-03	1,1E-03	6,6E-03
Shigella flexneri	7,3E-07	1,3E-05	3,9E-05	4,3E-05	2,5E-04
Klebsiella pneumoniae	1,4E-06	1,0E-05	6,0E-05	2,9E-05	9,8E-05
Salmonella enterica	6,5E-07	1,0E-05	4,7E-05	1,1E-05	3,6E-05
Enterobacter cloacae	1,5E-07	3,0E-06	8,6E-06	8,2E-06	3,5E-05

Supplementary Table 4

	Information							Mapping against FimH (LF82 as reference)		
Strain ID	Origine	Tissue / Source	Disease	Type of sequencing	Phylogroup quadruplex	Aggregation	%FimS ON	Presence of FimH	% identity LF82	bit score LF82
GI-AIEC02	GIRONA	Intestinal Biopsy	CD	MiSeq 300PE	C	Aggregation	25.0	Y	98.004	1567
GI-AIEC04	GIRONA	Intestinal Biopsy	Control	MiSeq 300PE	B2	Aggregation	20.0	Y	99.224	1628
GI-AIEC05	GIRONA	Intestinal Biopsy	CD	MiSeq 300PE	B2	Aggregation	0.0	Y	98.004	1567
GI-AIEC06	GIRONA	Intestinal Biopsy	Control	MiSeq 300PE	B2	Aggregation	34.8	Y	99.113	1622
GI-AIEC08	GIRONA	Intestinal Biopsy	Control	MiSeq 300PE	B2	Grey zone	8.0	Y	98.891	1611
GI-AIEC09	GIRONA	Intestinal Biopsy	CD	MiSeq 300PE	B2	Aggregation	21.9	Y	99.224	1628
GI-AIEC10	GIRONA	Intestinal Biopsy	Control	MiSeq 300PE	A	Aggregation	18.7	Y	97.783	1555
GI-AIEC11	GIRONA	Intestinal Biopsy	CD	MiSeq 300PE	E	Absence of aggregation	0.0	Y	99.667	1650
GI-AIEC12	GIRONA	Intestinal Biopsy	CD	MiSeq 300PE	C	Grey zone	0.0	Y	97.672	1550
GI-AIEC14_1	GIRONA	Intestinal Biopsy	CD	MiSeq 300PE	B2	Aggregation	26.7	Y	99.113	1622
GI-AIEC15_1	GIRONA	Intestinal Biopsy	CD	MiSeq 300PE	B2	Aggregation	51.8	Y	99.113	1622
GI-AIEC16_2	GIRONA	Intestinal Biopsy	CD	MiSeq 300PE	B2	Aggregation	9.1	Y	99.113	1622
GI-AIEC19	GIRONA	Intestinal Biopsy	Control	MiSeq 300PE	A	Absence of aggregation	0.0	Y	97.783	1555
GI-AIEC20	GIRONA	Intestinal Biopsy	CD	MiSeq 300PE	E	Absence of aggregation	0.0	Y	97.783	1555
GI-AIEC21	GIRONA	Intestinal Biopsy	CD	MiSeq 300PE	B2	Aggregation	4.3	Y	99.224	1628
GI-AIEC23	GIRONA	Intestinal Biopsy	CD	MiSeq 300PE	A	Aggregation	38.7	Y	97.783	1555
GI-AIEC24	GIRONA	Intestinal Biopsy	CD	MiSeq 300PE	C	Absence of aggregation	10.0	Y	98.004	1567
GI-AIEC25	GIRONA	Intestinal Biopsy	CD	MiSeq 300PE	B2	Grey zone	0.0	Y	100.000	1666
GI-ECG01	GIRONA	Intestinal Biopsy	CD	MiSeq 300PE	B2	Aggregation	13.6	Y	100.000	1666
GI-ECG02	GIRONA	Intestinal Biopsy	CD	MiSeq 300PE	B1	Absence of aggregation	0.0	Y	97.672	1550
GI-ECG04	GIRONA	Intestinal Biopsy	Control	MiSeq 300PE	B1	Aggregation	41.0	Y	98.115	1572
GI-ECG05	GIRONA	Intestinal Biopsy	CD	MiSeq 300PE	B2	Grey zone	0.0	Y	97.783	1555
GI-ECG09	GIRONA	Intestinal Biopsy	CD	MiSeq 300PE	B2	Absence of aggregation	0.0	Y	99.113	1622
GI-ECG15	GIRONA	Intestinal Biopsy	CD	MiSeq 300PE	B2	Absence of aggregation	5.4	Y	99.667	1650
GI-ECG16	GIRONA	Intestinal Biopsy	Control	MiSeq 300PE	C	Aggregation	0.0	Y	97.672	1550
GI-ECG17	GIRONA	Intestinal Biopsy	Control	MiSeq 300PE	B2	Aggregation	40.6	Y	100.000	1666
GI-ECG18	GIRONA	Intestinal Biopsy	CD	MiSeq 300PE	C	Absence of aggregation	0.0	N	NA	NA
GI-ECG19	GIRONA	Intestinal Biopsy	CD	MiSeq 300PE	E	Grey zone	11.8	Y	97.561	1544
GI-ECG21	GIRONA	Intestinal Biopsy	CD	MiSeq 300PE	B1	Aggregation	11.1	Y	97.672	1550

Supplementary Table 4

	Information							Mapping against FimH (LF82 as reference)		
**Strain ID**	Origine	Tissue / Source	Disease	Type of sequencing	Phylogroup quadruplex	Aggregation	%FimS ON	Presence of FimH	% identity LF82	bit score LF82
**GI-ECG26**	GIRONA	Intestinal Biopsy	CD	MiSeq 300PE	B2	Aggregation	11.1	Y	99.335	1633
**GI-ECG34**	GIRONA	Intestinal Biopsy	CD	MiSeq 300PE	E	Aggregation	11.8	Y	97.672	1550
**GI-ECG41**	GIRONA	Intestinal Biopsy	Control	MiSeq 300PE	B2	Grey zone	0.0	Y	99.224	1628
**GI-ECG42**	GIRONA	Intestinal Biopsy	CD	MiSeq 300PE	E	Aggregation	5.6	Y	97.561	1544
**GI-ECG43**	GIRONA	Intestinal Biopsy	Control	MiSeq 300PE	B2	Aggregation	22.2	Y	100.000	1666
**GI-ECG46**	GIRONA	Intestinal Biopsy	Control	MiSeq 300PE	B1	Absence of aggregation	0.0	N	NA	NA
**GI-ECG57**	GIRONA	Intestinal Biopsy	CD	MiSeq 300PE	E	Absence of aggregation	9.1	Y	97.783	1555
**GI-ECG63**	GIRONA	Intestinal Biopsy	CD	MiSeq 300PE	B1	Aggregation	35.7	Y	97.783	1555
**GI-ECG64**	GIRONA	Intestinal Biopsy	CD	MiSeq 300PE	B1	Aggregation	55.0	Y	98.115	1572
**GI-ECG65**	GIRONA	Intestinal Biopsy	CD	MiSeq 300PE	A	Absence of aggregation	5.6	Y	97.783	1555
**CU-08MY_1**	Cornell	Intestinal Biopsy	ICD	MiSeq 300PE	B2	Aggregation	9.5	Y	100.000	1666
**CU-09_396_A**	Cornell	Stool	Control	MiSeq 300PE	E	Aggregation	12.5	Y	97.561	1544
**CU-09_404_G**	Cornell	Stool	Control	MiSeq 300PE	B2	Aggregation	28.8	Y	100.000	1666
**CU-09_406_B**	Cornell	Stool	Control	MiSeq 300PE	C	Grey zone	0.0	Y	97.783	1555
**CU-09_407_A**	Cornell	Stool	Control	MiSeq 300PE	E	Absence of aggregation	0.0	Y	97.672	1550
**CU-09_410_C**	Cornell	Stool	Control	MiSeq 300PE	A	Absence of aggregation	6.9	Y	97.672	1550
**CU-09JW_1**	Cornell	Intestinal Biopsy	ICD	MiSeq 300PE	F	Absence of aggregation	4.8	Y	97.672	1550
**CU-09JW_2**	Cornell	Intestinal Biopsy	ICD	MiSeq 300PE	B1	Aggregation	35.1	Y	98.115	1572
**CU-13DK_1**	Cornell	Intestinal Biopsy	UC	MiSeq 300PE	B2	Absence of aggregation	0.0	Y	97.894	1561
**CU-13DK_9**	Cornell	Intestinal Biopsy	UC	MiSeq 300PE	B1	Absence of aggregation	4.3	Y	98.115	1572
**CU-14GK_1**	Cornell	Intestinal Biopsy	CCD	MiSeq 300PE	B1	Aggregation	0.0	Y	97.783	1555
**CU-15MH_1**	Cornell	Intestinal Biopsy	ICD	MiSeq 300PE	B1	Absence of aggregation	0.0	Y	97.672	1550
**CU-17LG_1**	Cornell	Intestinal Biopsy	H or non IBD	MiSeq 300PE	C	Absence of aggregation	0.0	Y	97.672	1550
**CU-18GB1**	Cornell	Intestinal Biopsy	ICD	MiSeq 300PE	B1	Absence of aggregation	20.0	Y	98.115	1572
**CU-19MD_1**	Cornell	Intestinal Biopsy	ICD	MiSeq 300PE	B2	Absence of aggregation	0.0	Y	97.894	1561
**CU-24LW_1**	Cornell	Intestinal Biopsy	ICD	MiSeq 300PE	A	Absence of aggregation	0.0	Y	98.004	1567
**CU-32SY_1**	Cornell	Intestinal Biopsy	ICD	MiSeq 300PE	B1	Aggregation	8.1	Y	98.115	1572
**CU-355_1**	Cornell	Intestinal Biopsy	CCD	MiSeq 300PE	E	Absence of aggregation	0.0	Y	99.667	1650
**CU-355_5**	Cornell	Intestinal Biopsy	CCD	MiSeq 300PE	B1	Aggregation	21.7	Y	97.783	1555

Supplementary Table 4

	Information							Mapping against FimH (LF82 as reference)		
**Strain ID**	Origine	Tissue / Source	Disease	Type of sequencing	Phylogroup quadruplex	Aggregation	%FimS ON	Presence of FimH	% identity LF82	bit score LF82
**CU-35MN_1**	Cornell	Intestinal Biopsy	H&non IBD	MiSeq 300PE	B1	Aggregation	33.3	Y	98.004	1567
**CU-35MN_4**	Cornell	Intestinal Biopsy	H&non IBD	MiSeq 300PE	E	Grey zone	9.4	Y	97.561	1544
**CU-37RT_1**	Cornell	Intestinal Biopsy	H&non IBD	MiSeq 300PE	C	Absence of aggregation	0.0	Y	97.894	1561
**CU-37RT_2**	Cornell	Intestinal Biopsy	H&non IBD	MiSeq 300PE	B2	Absence of aggregation	0.0	Y	97.783	1555
**CU-38AW_1**	Cornell	Intestinal Biopsy	ICD	MiSeq 300PE	B1	Aggregation	37.2	Y	98.115	1572
**CU-39ES_1**	Cornell	Intestinal Biopsy	ICD	MiSeq 300PE	B2	Absence of aggregation	0.0	Y	99.224	1628
**CU-40EM_1**	Cornell	Intestinal Biopsy	UC	MiSeq 300PE	A	Absence of aggregation	0.0	Y	97.672	1550
**CU-41CB_1**	Cornell	Intestinal Biopsy	ICD	MiSeq 300PE	B1	Absence of aggregation	0.0	Y	98.115	1572
**CU-41CB_2**	Cornell	Intestinal Biopsy	ICD	MiSeq 300PE	C	Absence of aggregation	0.0	N	NA	NA
**CU-42ET_1**	Cornell	Intestinal Biopsy	H&non IBD	MiSeq 300PE	B2	Grey zone	0.0	Y	99.667	1650
**CU-43FK_1**	Cornell	Intestinal Biopsy	H&non IBD	MiSeq 300PE	B1	Grey zone	3.1	Y	98.004	1567
**CU-470_1**	Cornell	Intestinal Biopsy	H&non IBD	MiSeq 300PE	F	Aggregation	0.0	Y	97.672	1550
**CU-48JD_1**	Cornell	Intestinal Biopsy	UC	MiSeq 300PE	B2	Aggregation	7.4	Y	100.000	1666
**CU-50PB_1**	Cornell	Intestinal Biopsy	H&non IBD	MiSeq 300PE	B2	Aggregation	0.0	Y	97.894	1561
**CU-524_2**	Cornell	Intestinal Biopsy	CCD	MiSeq 300PE	B1	Aggregation	8.1	Y	97.783	1555
**CU-524_6**	Cornell	Intestinal Biopsy	CCD	MiSeq 300PE	A	Absence of aggregation	0.0	Y	97.672	1550
**CU-52CS_1**	Cornell	Intestinal Biopsy	H&non IBD	MiSeq 300PE	C	Absence of aggregation	0	Y	97.894	1561
**CU-532_9**	Cornell	Intestinal Biopsy	ICD	MiSeq 300PE	B2	Aggregation	34.1	Y	99.557	1644
**CU-538_10**	Cornell	Intestinal Biopsy	ICD	MiSeq 300PE	F	Aggregation	7.4	Y	98.448	1589
**CU-538_6**	Cornell	Intestinal Biopsy	ICD	MiSeq 300PE	B1	Absence of aggregation	0.0	Y	98.004	1567
**CU-541_1**	Cornell	Intestinal Biopsy	ICD	MiSeq 300PE	B1	Absence of aggregation	0.0	Y	97.894	1561
**CU-541_15**	Cornell	Intestinal Biopsy	ICD	MiSeq 300PE	A	Absence of aggregation	0.0	Y	97.672	1550
**CU-545_4**	Cornell	Intestinal Biopsy	ICD	MiSeq 300PE	C	Absence of aggregation	0.0	N	NA	NA
**CU-546_1**	Cornell	Intestinal Biopsy	ICD	MiSeq 300PE	B2	Aggregation	18.2	Y	98.448	1589
**CU-54AR_1**	Cornell	Intestinal Biopsy	H&non IBD	MiSeq 300PE	A	Absence of aggregation	3.8	Y	97.672	1550
**CU-552_2**	Cornell	Intestinal Biopsy	ICD	MiSeq 300PE	B1	Aggregation	0.0	Y	97.783	1555
**CU-55AS_1**	Cornell	Intestinal Biopsy	H&non IBD	MiSeq 300PE	C	Aggregation	0.0	Y	97.672	1550
**CU-568_3**	Cornell	Intestinal Biopsy	CCD	MiSeq 300PE	B2	Absence of aggregation	0.0	N	NA	NA

Supplementary Table 4

	Information							Mapping against FimH (LF82 as reference)		
**Strain ID**	Origine	Tissue / Source	Disease	Type of sequencing	Phylogroup quadruplex	Aggregation	%FimS ON	Presence of FimH	% identity LF82	bit score LF82
**CU-576_1**	Cornell	Intestinal Biopsy	ICD	MiSeq 300PE	E	Grey zone	5.9	Y	97.783	1555
**CU-576_10**	Cornell	Intestinal Biopsy	ICD	MiSeq 300PE	E	Grey zone	3.0	Y	97.783	1555
**CU-578_1**	Cornell	Intestinal Biopsy	ICD	MiSeq 300PE	E	Grey zone	0.0	Y	97.783	1555
**CU-584_1**	Cornell	Intestinal Biopsy	CCD	MiSeq 300PE	C	Absence of aggregation	0.0	Y	98.004	1567
**CU-589_1**	Cornell	Intestinal Biopsy	UC	MiSeq 300PE	B2	Absence of aggregation	0.0	Y	97.894	1561
**CU-58PP_1**	Cornell	Intestinal Biopsy	UC	MiSeq 300PE	B2	Aggregation	2.4	Y	97.894	1561
**CU-601_1**	Cornell	Intestinal Biopsy	H&non IBD	MiSeq 300PE	B1	Absence of aggregation	0.0	N	NA	NA
**CU-602_1**	Cornell	Intestinal Biopsy	UC	MiSeq 300PE	B2	Absence of aggregation	0.0	Y	98.004	1567
**CU-603_1**	Cornell	Intestinal Biopsy	ICD	MiSeq 300PE	A	Absence of aggregation	0.0	Y	97.453	1539
**CU-60CM_1**	Cornell	Intestinal Biopsy	UC	MiSeq 300PE	F	Absence of aggregation	0.0	Y	97.894	1561
**CU-73EL_1**	Cornell	Intestinal Biopsy	ICD	MiSeq 300PE	B1	Aggregation	30.9	Y	97.672	1550
**CU-TI_1**	Cornell	Intestinal Biopsy	ICD	MiSeq 300PE	C	Absence of aggregation	0.0	Y	97.894	1561

Supplementary Figure 1

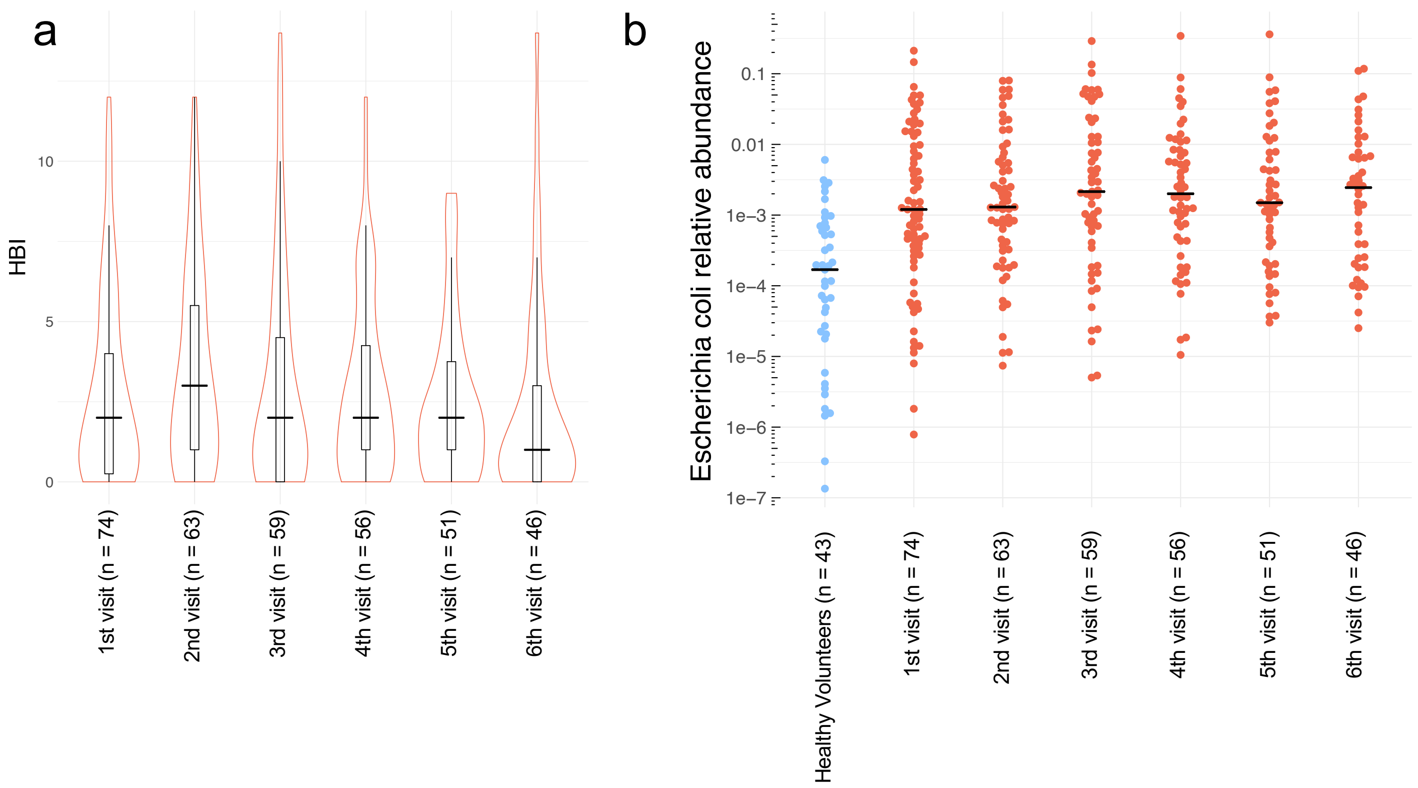

Supplementary Figure 2

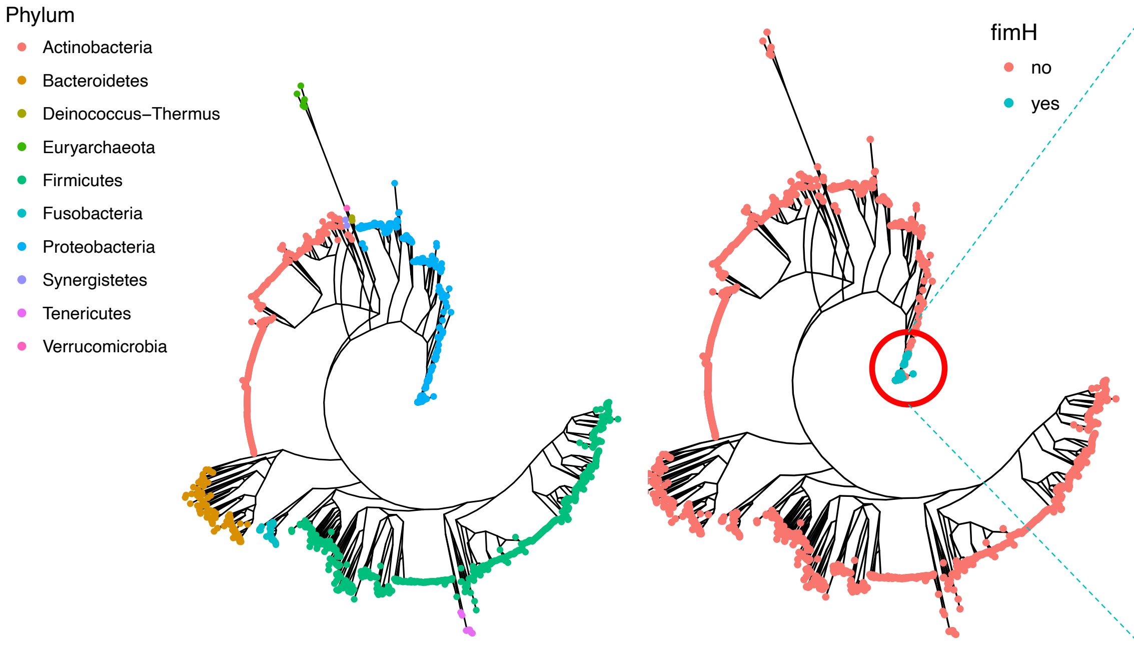

**Species**
Citrobacter farmeri
Citrobacter rodentium
Enterobacter aerogenes
Escherichia alberti
Escherichia coli
Klebsiella pneumoniae
Klebsiella quasipneumoniae
Klebsiella variicola
Kluyvera ascorbata
Providencia stuartii
Raoultella ornithinolytica
Raoultella planticola
Salmonella enterica
Shigella boydii
Shigella dysenteriae
Shigella flexneri
Shigella sonnei
Yersinia fredericksenii
Yersinia intermedia

Supplementary Figure 3

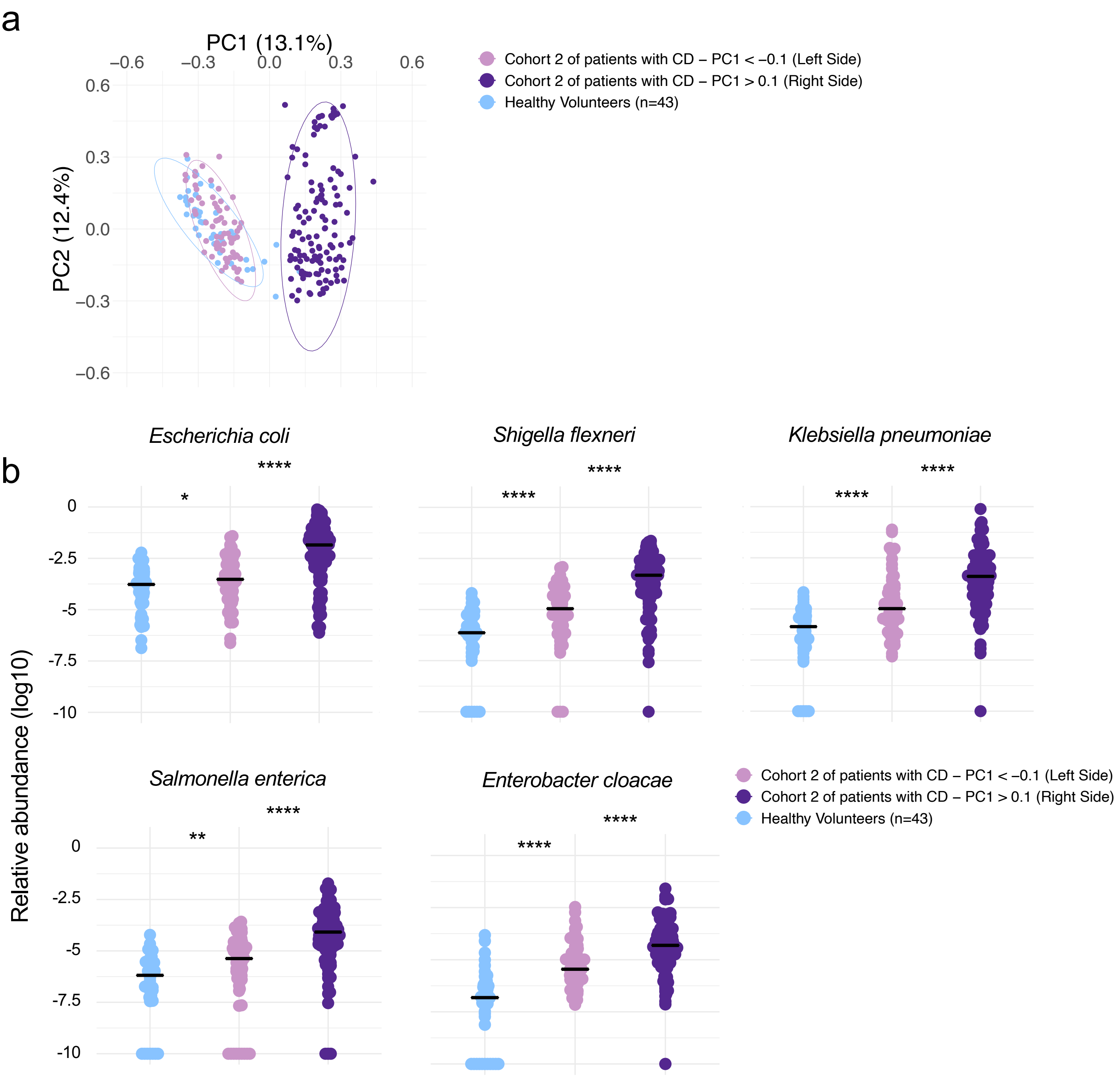

Supplementary Figure 4

TAK-018

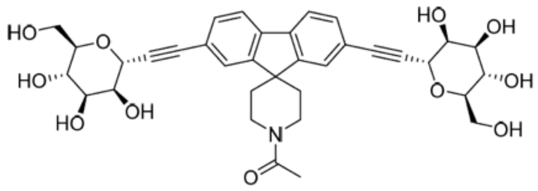

Supplementary Figure 5

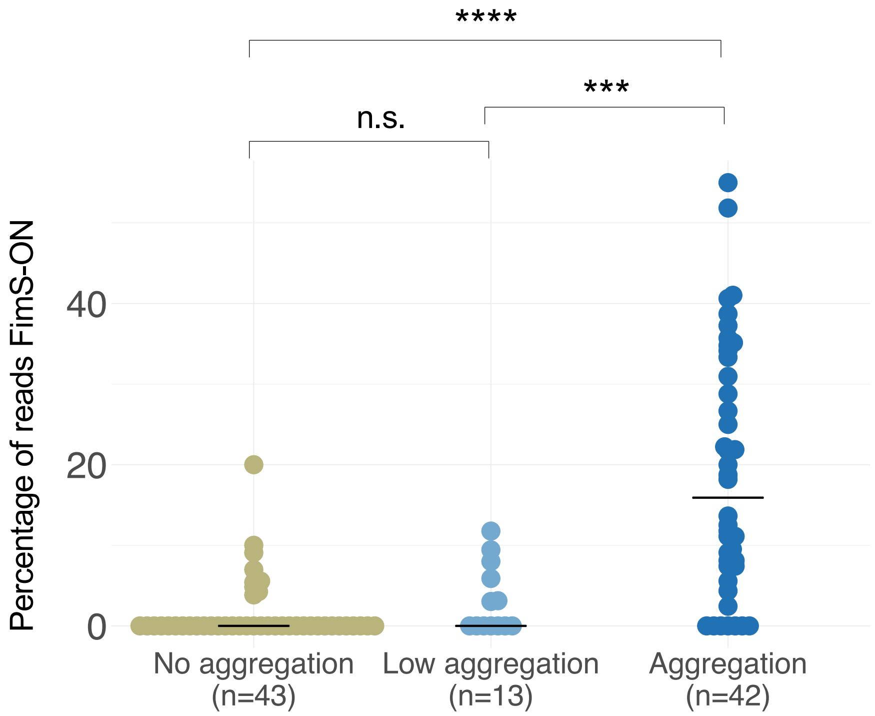

Supplementary Figure 6

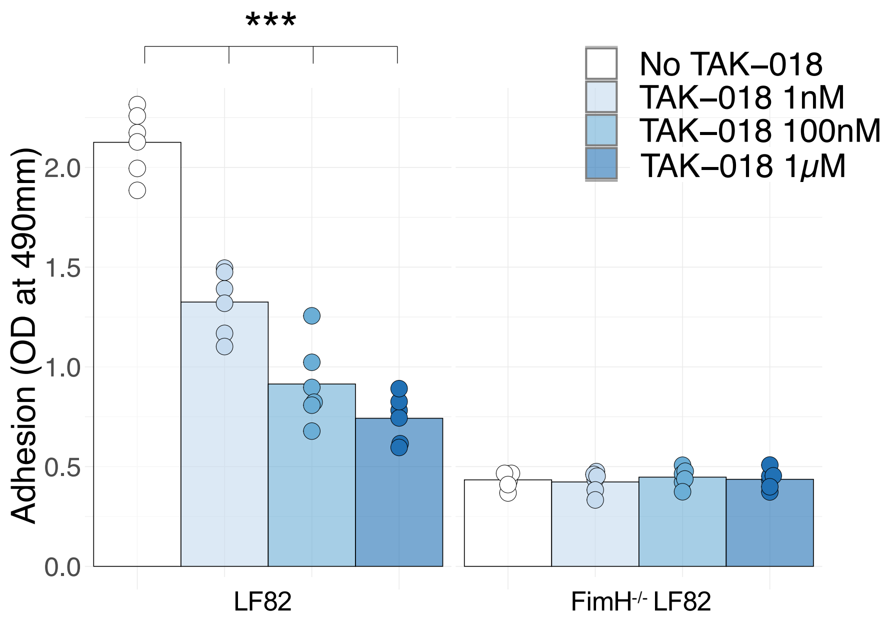

Supplementary Figure 7

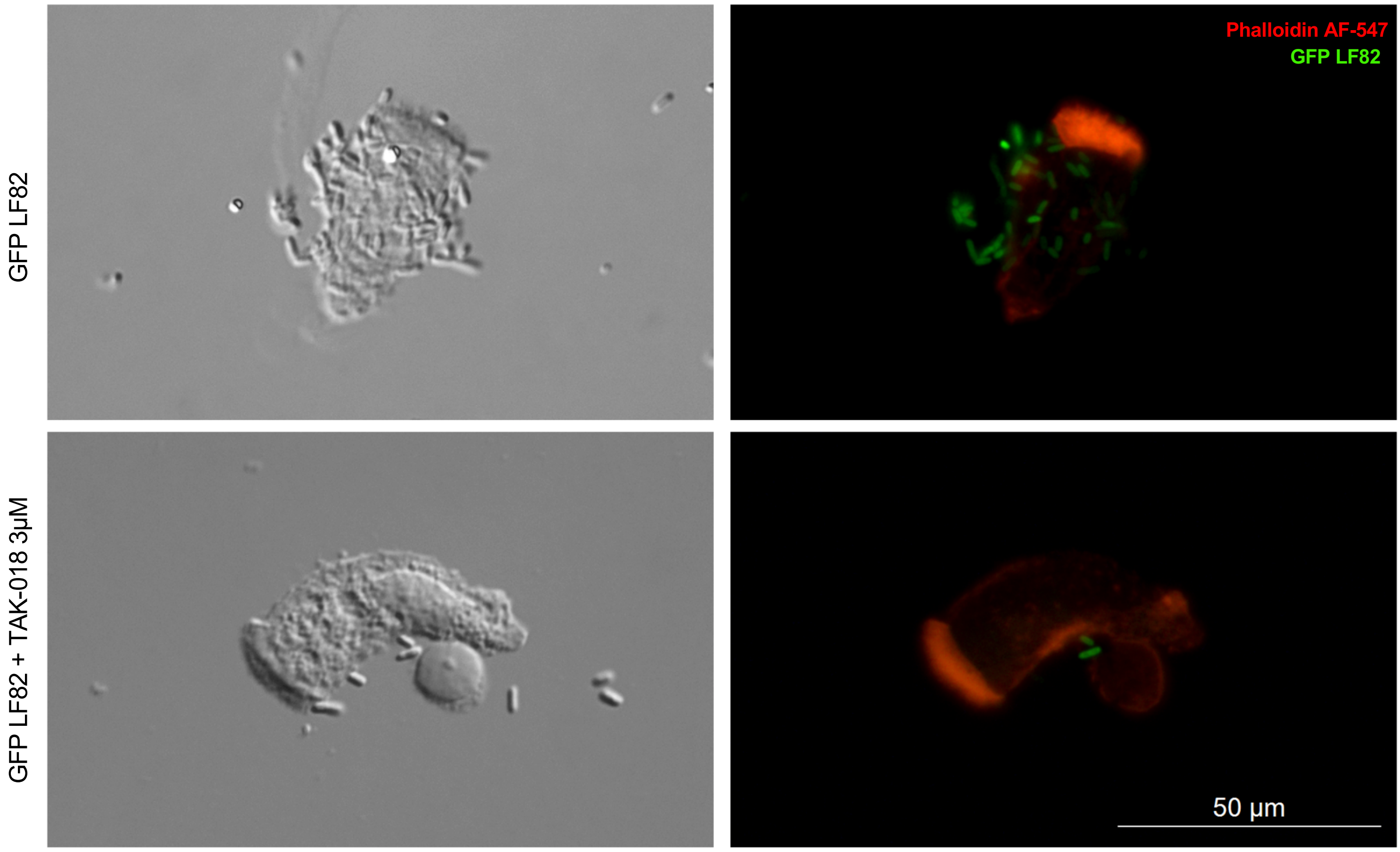

Supplementary Figure 8

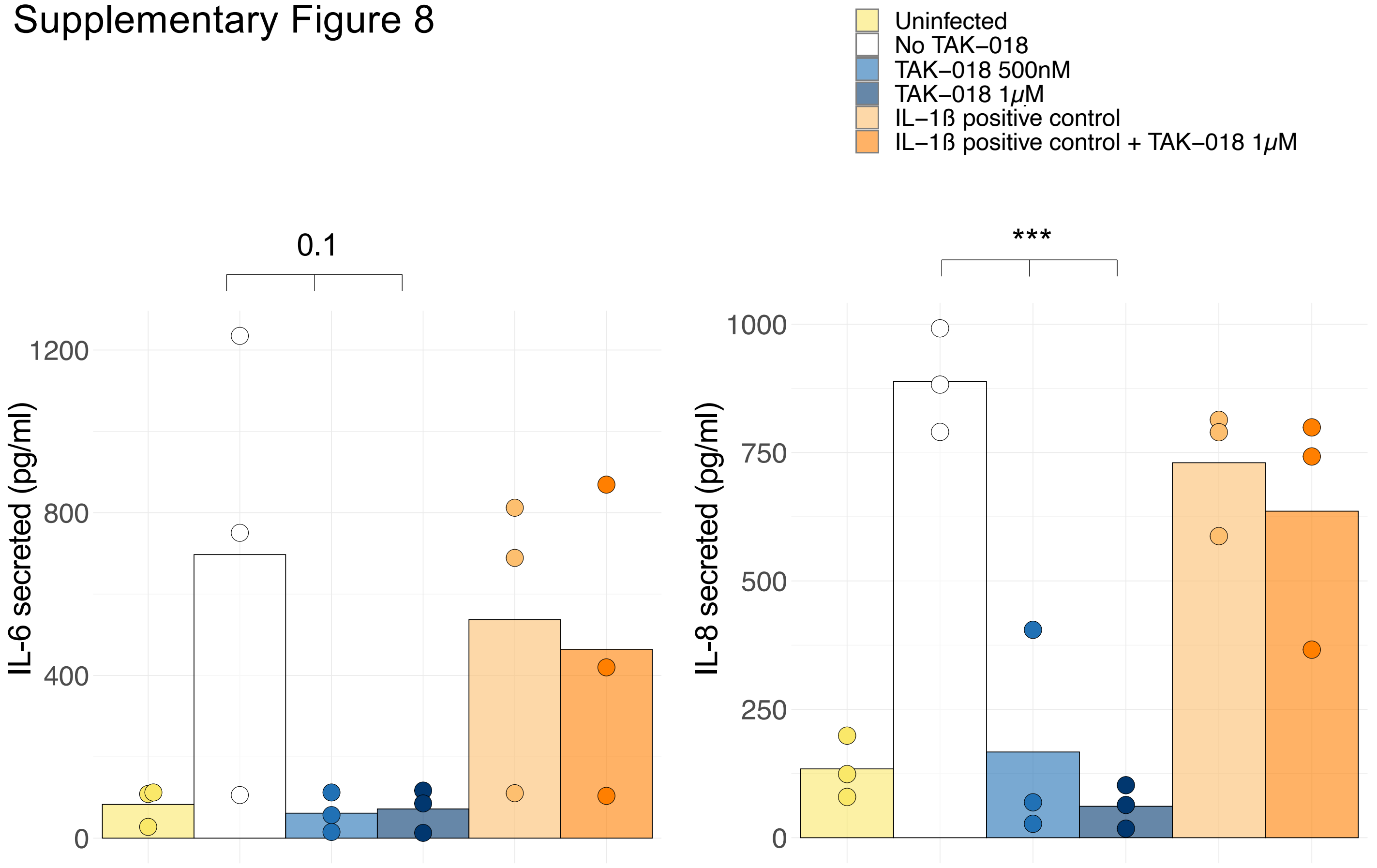

Supplement: Supplementary file 2 — Additional file 1: Supplementary Table 1. Demographic data and history of Crohn’s Disease of the patients with CD included in each of the presented studies. *: Montreal classification for Cohort 1 and 3. **: MOBIDIC: QUANTA Lite/LLOD=15.6mg/kg. CrohnOmeter: Bühlmann/LLOD=50mg/kg. PREDICT: Bühlmann/LLOD=30mg/kg. NA=Not available. Supplementary Table 2. Demographic data of the healthy volunteers included in the presented study. Supplementary Table 3. Median values for Enterobacteriaceae species in Fig. 2c (top table) and d (bottom table). Supplementary Table 4. List of isolated E. coli strains and associated information. Supplementary Figure 1. Evolution over time of the first cohort regarding HBI (left) and E. coli relative abundance (right). A linear mixed model was used to identify statistically significant differences between time points (visits) and did not reach significance for E. coli abundance (P = 0.51) nor for HBI (P = 0.41). Supplementary Figure 2. Some Proteobacteria express FimH adhesin. Cladogram representing the bacteria phyla detected in the human gut microbiome (left) and FimH presence with a focus on Enterobacteriaceae spp (right). Supplementary Figure 3. Dichotomy in patient with CD from Cohort 2. a, Microbial clustering as shown based on Bray–Curtis dissimilarity principal Coordinate Analysis (PCoA) metrics for HV and patients with CD from Cohort2 with PC1 ≤ 0.1 and PC1 > 0.1. Ellipsoids represent a 95% confidence interval surrounding each group. b, Relative abundance of Enterobacteriaceae spp in HV and patients with CD from Cohort2 with PC1 ≤ 0.1 and PC1 > 0.1. Non-parametric Mann-Whitney U test was used to identify the statistically significant differences between groups. (* P < 0.05, ** P < 0.005, **** P < 0.0001). Supplementary Figure 4. Structure of the bi-mannosylated FimH-blocker TAK-018. Supplementary Figure 5. Association between percentage of FimS-ON expression and aggregation to TAK-018 of different AIEC strains. The mapping against the [file 40168_2021_1135_MOESM2_ESM.pdf]
